# Insights into the complex role of GRAS transcription factors in the arbuscular mycorrhiza symbiosis

**DOI:** 10.1038/s41598-019-40214-4

**Published:** 2019-03-04

**Authors:** Rico M. Hartmann, Sieke Schaepe, Daniel Nübel, Arne C. Petersen, Martina Bertolini, Jana Vasilev, Helge Küster, Natalija Hohnjec

**Affiliations:** 1 Unit IV-Plant Genomics, Institute of Plant Genetics, Leibniz Universität Hannover, Herrenhäuser Str. 2, D-30419 Hannover, Germany; 2 Department of Food, Environmental and Nutritional Sciences, Università degli Studi di Milano, Via Mangiagalli 25, 20133 Milano, Italy

## Abstract

To improve access to limiting nutrients, the vast majority of land plants forms arbuscular mycorrhizal (AM) symbioses with Glomeromycota fungi. We show here that AM-related GRAS transcription factors from different subgroups are upregulated during a time course of mycorrhization. Based on expression studies in mutants defective in arbuscule branching (*ram1-1*, with a deleted *MtRam1* GRAS transcription factor gene) or in the formation of functional arbuscules (*pt4-2*, mutated in the phosphate transporter gene *MtPt4*), we demonstrate that the five AM-related GRAS transcription factor genes *MtGras1*, *MtGras4*, *MtGras6*, *MtGras7*, and *MtRad1* can be differentiated by their dependency on MtRAM1 and MtPT4, indicating that the network of AM-related GRAS transcription factors consists of at least two regulatory modules. One module involves the MtRAM1- and MtPT4-independent transcription factor MtGRAS4 that activates *MtGras7*. Another module is controlled by the MtRAM1- and MtPT4-dependent transcription factor MtGRAS1. Genome-wide expression profiles of mycorrhized *MtGras1* knockdown and *ram1-1* roots differ substantially, indicating different targets. Although an *MtGras1* knockdown reduces transcription of AM-related GRAS transcription factor genes including *MtRam1* and *MtGras7*, *MtGras1* overexpression alone is not sufficient to activate *MtGras* genes. *MtGras1* knockdown roots display normal fungal colonization, with a trend towards the formation of smaller arbuscules.

## Introduction

Plants are able to form mutualistic associations with microbial soil organisms to their own benefit. The symbiosis with arbuscular mycorrhizal (AM) fungi of the *Glomeromycota*^1^ can be found in more than 80% of all land plants^2^. While the fungal symbiont is supplied with photosynthetically fixed carbon, its widespread mycelial network expands the phosphate depletion zone of the rhizosphere and actively provides phosphorus, but also other nutrients, to the host plant^3^.

Emerging from the hyphopodium that extraradical hyphae form on the root surface, a structure called prepenetration apparatus (PPA^4^) is established in epidermal cells. The PPA guides hyphal growth towards the inner cortex, where fungal hyphae proliferate^5^, ultimately leading to the formation of tree-like intracellular arbuscules^6,7^. These symbiotic structures are regarded as the central place of nutrient transfer between plant cells and AM fungi^8,9^. In return for the supply of photosynthetically fixed carbon to the microsymbiont^10,11^, the AM fungus primarily delivers phosphorus, but also nitrogen compounds, minerals, and water across the periarbuscular membrane to the host plant^12,13^. This plant-derived, highly specialized interface is thus equipped with a specific composition of transporters and other membrane-associated proteins^10^.

Arbuscules are transient structures that only operate for a couple of days^14^, and a suite of AM-activated transcriptional regulators belonging to different classes^15–19^ controls their development, functionality, and degradation. The regular turnover of arbuscules is initiated by a senescence program^20^ that involves the MtMYB1-induced activation of genes encoding hydrolases and defense-related proteins, together supporting the cellular restructuring of arbuscule-containing cells^21^.

Transcription factors (TFs) can be found in all eukaryotic organisms. Functioning as regulators of gene expression that interact with enhancer regions of promoters to induce or repress transcription of target genes, they control both plant development and its reactions to external abiotic and biotic stimuli. The higher number of plant TFs in comparison to animals implies an involvement in the continuous adaption of plants to the environment, which cannot be avoided due to their sessile nature^22,23^.

GRAS transcription factors belonging to the *GIBBERELLIN-INSENSITIVE (GAI*^24^), *REPRESSOR of gal1-3 (RGA*^25^), or *SCARECROW (SCR*^26^) families form a subgroup of plant TFs. Based on their specific domains, 59^27^ or 68^28^ members of the GRAS TF family were predicted in *M*. *truncatula*. Prominent GRAS TFs have been shown to play a vital role in symbiotic signaling^29^, e.g. NSP1 and NSP2 that have a key role in the early transduction of signals during rhizobial and mycorrhizal symbioses. In response to the elicitation by Nod-factors (NFs), NSP1 and NSP2 form a heterodimer that binds to *cis*-regulatory elements in the promoter of the *ENOD11* gene^30^. NSP1 and NSP2 also mediate other early Nod- and also Myc-factor induced responses, a process that incorporates the GRAS TF RAM1^31,32^. RAM1 was initially shown to be required for early mycorrhizal signaling^31^, but is now known to control arbuscule branching in *Medicago truncatula*, *Lotus japonicus*, and *Petunia hybrida*^33–36^. Transcription profiling of *ram1-1* mutants in pre-symbiotic signaling^37^ and in AM roots^16^ revealed several hundred potential targets of RAM1, including many members of the carbohydrate and lipid metabolism^16^. In *M*. *truncatula*, a major task of this GRAS TF is the induction of the *MtRam2* gene, encoding a glycerol-3-phosphate acyltransferase involved in the production of fatty acid precursors required for the formation of the periarbuscular membrane^38^. In addition, MtRAM1 is required for the expression of the AM-induced phosphate transporter gene *MtPt4* and genes encoding other membrane transporters and membrane proteins essential for arbuscule function^33^. Downstream targets of RAM1 also include genes of the *WRI* family (*MtWRI5a*, *MtWRI5b*, and *MtWRI5c* in *M*. *truncatula*^39^ and *CBX1* in *Lotus japonicus*^40^), all encoding AP2-domain TFs that regulate genes related to fatty acid biosynthesis. Expression of *MtRam1* and *MtWri5a* was shown to be interdependent, forming a regulatory feedback loop between the encoded TFs^39^.

The activation of *MtRam1* transcription^34^ is controlled by DELLA proteins, which form a subgroub of GRAS-TFs. DELLA proteins, being inactivated at high GA levels, were thus shown to link the level of plant hormones with arbuscule formation^20,41^.

RAD1, a second prominent member of the AM-related GRAS TF family, was in addition to RAM1 shown to be required for arbuscule development in *M*. *truncatula*^42^ and *L*. *japonicus*^36^. Interestingly, RAD1 was also shown to interact with RAM1^36^ as well as TF80 and TF124, two additional AM-related GRAS TFs^33^, suggesting that these regulators interact to control arbuscule development^33,36^.

A mycorrhiza-inducible clade of GRAS TFs (MIG) has recently been shown to be already activated by *Rhizophagus* spore exudates. Amongst these TFs, MtMIG1 was shown to be crucial for radial cell expansion and arbuscule development by interacting with MtDELLA1, thus intersecting the GA-pathway in mycorrhizal roots^43^.

GRAS TFs have not only been reported to separately control regulatory processes during mycorrhization^33,43^. Several studies have in addition identified a direct interaction of GRAS proteins e.g. MtNSP1-MtNSP2^30^, MtRAM1-MtTF80^33^, MtRAM1-MtRAD1^33^, MtRAM1-MtNSP2^31^, MtRAD1-MtNSP2^43^, MtMIG1-NSP1^43^, and MtMIG1-DELLA1^43^, supporting the idea that networks of GRAS TFs interact to control mycorrhization and in particular arbuscule development^17^.

Based on genome-wide expression profiling of mycorrhizal and non-mycorrhizal tissues^44–49^, several *M*. *truncatula* genes encoding GRAS TFs (*MtGras* genes) were found to be upregulated in AM symbioses, including the *MtRad1*^42^ and *TF80* genes^33^ mentioned above. For our study, we selected both those *MtGras* genes exclusively activated during mycorrhization and those that were AM-induced but not AM-specific, since they are also expressed in non-symbiotic conditions or in different tissues. To shed light on the contribution of the AM-activated *MtGras* family to the development of AM symbioses and arbuscule formation, we performed comparative gene expression studies and *in situ* localizations of promoter activities in wild type plants and in the *ram1-1* mutant lacking a key transcriptional regulator of arbuscule branching^33^ as well as the *pt4-2* mutant^50^, characterized by a defective phosphate transporter required for the formation of active, phosphate-transporting arbuscules. Together with functional studies in either *Tnt1* mutants or RNAi-mediated knockdown roots, we propose a model where we position five AM-related GRAS TFs relative to the well-studied AM-related regulator MtRAM1 in the regulatory circuit that controls arbuscule development. With MtGRAS1, we provide evidence that an AM-related GRAS TF is part of a feedback loop with MtRAM1 to sustain arbuscule formation.

## Results

### AM-related GRAS TFs of *Medicago truncatula* belong to different subgroups

**Figure 1:**
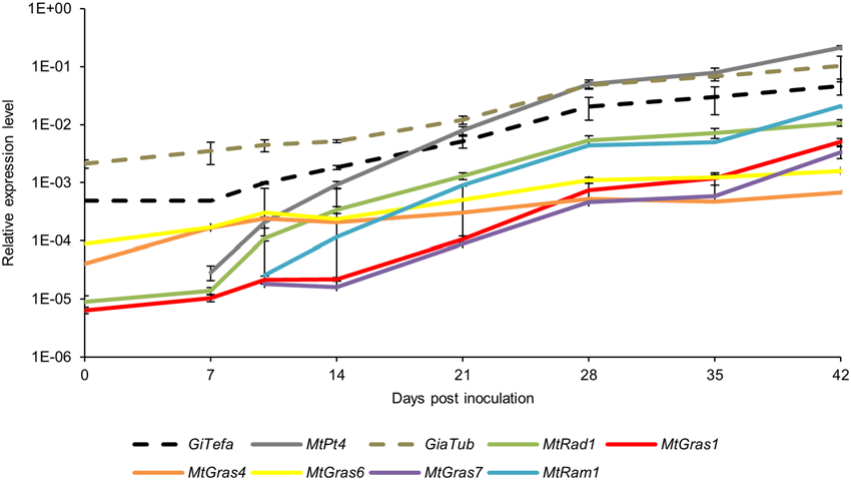
Time course of AM-responsive gene expression. 28 days after germination, plants were mycorrhized and harvested at the time points indicated. Plants at 0 days post inoculation were harvested after 3 h of inoculation with *R*. *irregularis* spores. At each time point, three biological replicates were harvested, each consisting of 6 pooled root systems. Relative gene expression levels were determined by real-time RT-PCR. The standard error of the mean is indicated.

Based on genome-wide expression profiles recorded by GeneChip hybridizations^47^, a core set of AM-activated GRAS TF genes, namely *MtGras1* (designated *TF80* in^33^), *MtGras4*, *MtGras6*, *MtGras7*, and *MtRad1*^42^ was selected. In addition, the *MtRam1* gene^31^, encoding a GRAS TF that controls arbuscule branching^33^, was included. The corresponding identifiers from the *Medicago truncatula* genome^51^, the *Medicago* Gene Expression Atlas^52^, and the literature are listed in Supplementary Table S1. Analyses of gene expression data from GeneChip hybridizations stored in the Medicago Gene Expression Atlas^52^ and from studies on *MtRam1*^31^ revealed two different subgroups among the selected *MtGras* genes. Whereas *MtGras1*, *MtGras7*, *MtRad1*, and *MtRam1* are specifically expressed in mycorrhizal roots and are only activated at background levels in non-mycorrhized controls, *MtGras4* and *MtGras6* display a low but detectable expression in non-mycorrhizal roots (Supplementary Fig. S1), yet showing a significant upregulation upon mycorrhization^47^. This corresponds to the data shown in Fig. 1, where *MtGras4* and *MtGras6* are expressed at significant levels at time point 0. Although being AM-induced, *MtGras4* and *MtGras6* transcription also occurs in other tissues^52^. We nevertheless selected these genes in addition to the AM-specific *MtGras1*, *MtGras7*, *MtRad1*, and *MtRam1* genes, because arbuscule formation responds to a range of nutritional, physiological, and environmental factors^16^ that obviously are relevant in non-symbiotic tissues as well.

In a phylogenetic tree of the deduced amino acid sequences of these and selected other symbiotic GRAS TF genes, specific groupings were evident (Supplementary Fig. S2). Interestingly, MtGRAS4 and MtGRAS7, two GRAS TFs belonging to the MIG1 family defined by^43^ share the highest sequence similarities, suggesting a functional relationship although only *MtGras7* is specifically expressed in AM roots^47^. In addition, the close relation of MtRAM1 and MtRAD1 is of special interest, since these TFs control arbuscule branching as well as arbuscule development and since mutual protein-protein interactions were reported^33,36^.

### AM-related GRAS TF genes are differentially upregulated in the course of mycorrhization

To reveal the timing of GRAS TF action during the development of an AM symbiosis, a gene expression time course study was performed (Fig. 1). Histological analyses of the harvested mycorrhizal roots indicated the predominant presence of *R*. *irregularis* spores and extraradical hyphae until 7 days post inoculation (dpi), while significant intraradical colonization and arbuscule development started from 10 dpi.

Since root material at 0 dpi already contained germinated *R*. *irregularis* spores, the AM fungal marker genes *GiTefa* (encoding a translation elongation factor alpha) and *GiaTub* (encoding an *a*-tubulin) are already expressed, whereas transcription of *MtPt4*, encoding an arbuscule-specific phosphate transporter^50^, is only detected upon arbuscule presence. While the expression of *GiTefa* and *GiaTub* showed a linear increase over time, *MtPt4* activation rose almost exponentially, mirroring the quick and ongoing process of arbuscule build-up.

During mycorrhization, the transcriptional activation of *MtGras4* and *MtGras6*, being upregulated appr. 16- to 17-fold between 0 dpi and 42 dpi (Fig. 1) resembled that of *GiaTub* and *GiTefa*, whereas *MtGras1*, *MtGras7*, *MtRad1*, and *MtRam1* (appr. 180- to 1200-fold upregulation from the time point of their first expression, Fig. 1) followed the strong rise in *MtPt4* transcription. These patterns indicate two different types of activation, with *MtGras4* and *MtGras6* being already markedly expressed at 0 dpi, probably due to their weak expression in non-mycorrhized roots (*Medicago* Gene Expression Atlas^52^ and^47^), while the upregulation of *MtGras1*, *MtGras7*, *MtRad1*, and *MtRam1* follows the ongoing build-up of functional arbuscules in *MtPt4*-expressing cells.

### AM-related GRAS TF genes differ in their dependency on the GRAS TF MtRAM1

To study the dependency of AM-related GRAS TF gene expression on MtRAM1, a GRAS TF required for arbuscule branching^33^, comparative gene expression analyses were carried out in *R*. *irregularis*-mycorrhized roots of wild type plants and *ram1-1* mutants.

**Figure 2:**
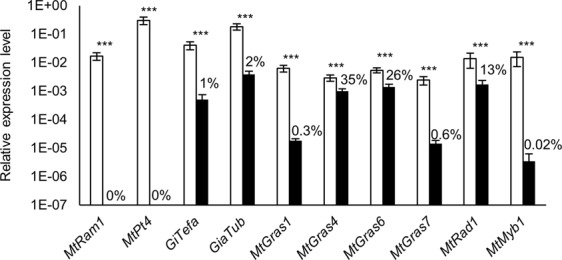
Relative expression of selected *MtGras* and AM marker genes in mycorrhized *M*. *truncatula* A17 wild type (white) and *ram1-1* (black) roots. Transcript amounts are shown relative to *MtTefα*. Roots were harvested at 36 days post inoculation with *R*. *irregularis*. n = 8 biological replicates, error bars represent standard deviations. Numbers indicate the percental expression level compared to the wild type. The following genes were analyzed in addition to the *MtGras* genes listed in Supplementary Table S1: *MtRam1*^33^, *MtPt4*^9^, *GiTefα*^69^, *GiαTub*^70^, *MtRad1*^42^, and *MtMyb1*^18^. ***p < 0.001 (Student’s t-test).

Whereas real-time RT-PCR measurements (Fig. 2) of mycorrhizal roots showed a strong decrease in fungal gene expression (*GiTefa*, *GiaTub*; down to 1–2%) as well as a complete lack of *MtRam1* and *MtPt4* transcription in *ram1-1* mutants, indicating the absence of highly branched, symbiotically active arbuscules, the reduction of GRAS TF gene transcription appeared diverging. While expression of *MtGras1* and *MtGras7* strongly decreased to 0.3–0.6% of the level in wild type roots, transcription of *MtRad1*, *MtGras4*, and *MtGras6* was less affected (reduction to 13–35%). A strongly decreased expression in *ram1-1* mutants was also observed for the AM-specific *MtMyb1* gene, encoding a key regulator of arbuscule degradation^18^.

Since fungal colonization is impaired in *ram1-1* mutants and since expression of AM-induced marker genes as well as *MtGras1* and *MtRad1* was lower in *ram1-1* mutants in response to colonization by *Glomus versiforme*^33^, a reduced expression of the AM-induced GRAS TF genes studied here was expected. Thus, expression levels of the genes studied (Fig. 2) were divided by the *GiTefa* transcription level in order to adjust gene expression to the amount of fungal tissue. This procedure revealed that lower transcription levels of *MtGras1* (24.3%), *MtGras7* (0.01%), and *MtMyb1* (0.03%) were still evident in *R*. *irregularis* colonized *ram1-1* in comparison to wild type roots, indicating that the expression of these genes is not just reduced in *ram1-1* mutants as a consequence of lower fungal colonization.

**Figure 3:**
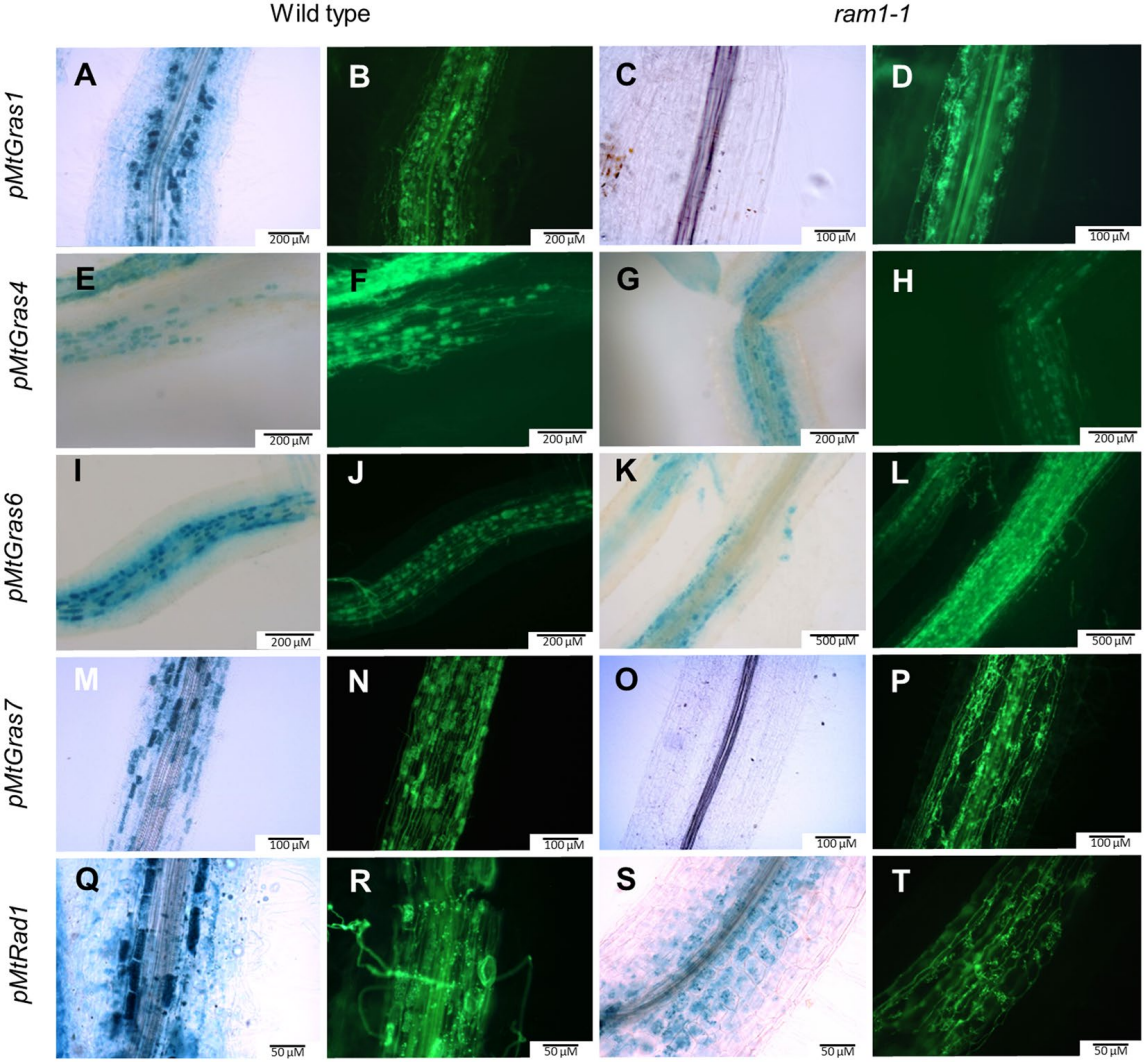
Histochemical localization of the promoter activities of selected *MtGras* genes. The promoter activities of *MtGras1* (**A–D**), *MtGras4* (**E–H**), *MtGras6* (**I–L**), *MtGras7* (**M–P**), *and MtRad1* (**Q–T**) were recorded in transgenic, mycorrhized roots of *M*. *truncatula* A17 wild type (**A**,**B**,**E**,**F**,**I**,**J**,**M**,**N**,**Q**,**R**) and *ram1-1* roots (**C**,**D**,**G**,**H**,**K**,**L**,**O**,**P**,**S**,**T**). GUS stainings were performed for 4–8 hours. Alexa WGA Fluor 488 stainings are shown to visualize fungal colonization.

To achieve a cellular resolution of GRAS TF gene expression, their promoter regions were fused to the *gusA*int reporter gene, and the resulting transcriptional fusions were expressed in transgenic *M*. *truncatula* roots. These studies demonstrated a clearly AM-induced promoter activity for *MtGras1*, *MtGras4*, *MtGras6*, *MtGras7*, and *MtRad1* in wild type roots, with a predominant or exclusive activation in the arbuscule-containing cells (Fig. 3). In *ram1-1* mutants, promoter activities of *MtGras1* and *MtGras7* were completely abolished, even after prolonged staining. In contrast, *MtGras4*, *MtGras6*, and *MtRad1* promoters are still AM-induced in the *ram1-1* mutant background. These findings were in line with our gene expression studies (Fig. 2) and suggested a position of MtGRAS1 and MtGRAS7 downstream of MtRAM1 in the regulatory cascade leading to arbuscule formation, while MtGRAS4, MtGRAS6, and MtRAD1 have to be placed either upstream or parallel to MtRAM1.

### Expression of AM-related GRAS TF genes differs in the dependency on morphologically fully developed arbuscules

To study the dependency of GRAS TF gene expression on the presence of functional, phosphate-transporting arbuscules, comparative gene expression analyses were carried out in *R*. *irregularis*-mycorrhized roots of wild type plants and *pt4-2* mutants.

**Figure 4:**
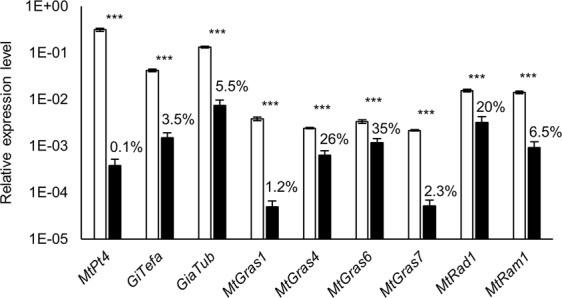
Relative expression of selected *MtGras* and AM marker genes in mycorrhized *M*. *truncatula* A17 wild type (white) and *pt4-2* (black) roots. Transcript amounts are shown relative to *MtTefα*. Roots were harvested at 36 days post inoculation with *R*. *irregularis*. n = 8 biological replicates, error bars represent standard deviations. Numbers indicate the percental expression level compared to the wild type. Genes shown are defined in the Fig. 2 legend. ***p < 0.001 (Student’s t-test).

Real-time RT-PCR analyses of *pt4-2* mutants revealed a significant regulation of the GRAS TF genes investigated similar to *ram1-1* mutants (Fig. 4). In addition to *MtPt4*, the *MtGras1* and *MtGras7* genes are most strongly repressed in the *pt4-2* background (to 1.2–2.3% of wild type expression), whereas other GRAS TF genes were less strongly affected. After a division by the *GiTefa* expression level, only *MtGras1* and *MtGras7* transcription was reduced to 36.9% and 66.6% in *pt4-2* in comparison to wild type roots, respectively, indicating that this downregulation is not just reflecting a lower degree of colonization in *pt4-2* roots.

**Figure 5:**
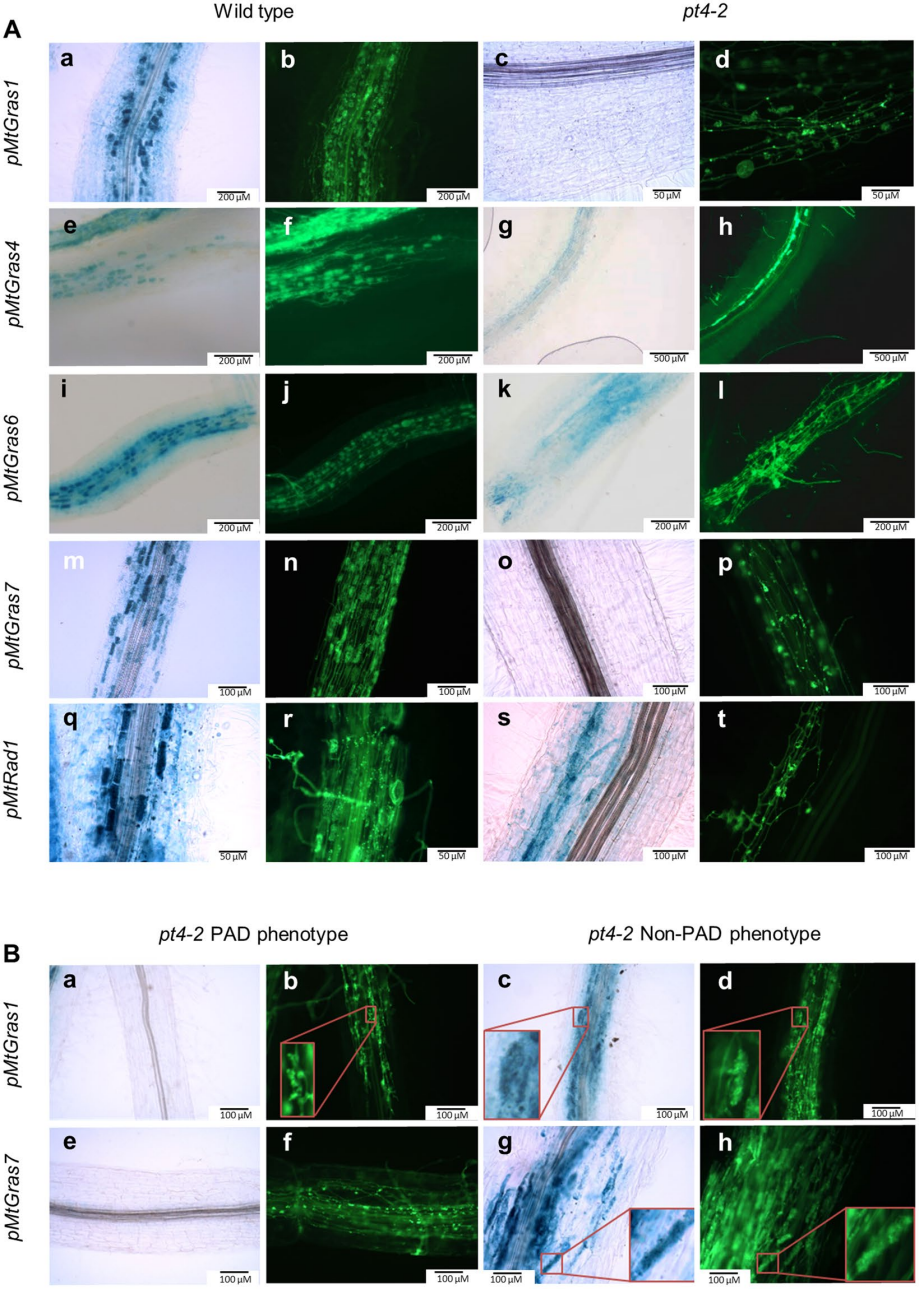
Histochemical localization of the promoter activities of selected *MtGras* genes. The promoter activities of *MtGras1* (**A**: a–s), *MtGras4* (**A**: e–h), *MtGras6* (**A**: i–l), *MtGras7* (**A**: m–p), *and MtRad1* (**A**: q–t) were recorded in transgenic, mycorrhized roots of *M*. *truncatula* A17 wild type (**A**: a,b,e,f,i,j,m,n,q, and r) and *pt4-2* roots (c,d,g,h,k,l,o,p,s, and t). The promoter activities of *MtGras1* (**B**: a–d) and *MtGras7* (**B**: e–h) were furthermore recorded in transgenic, mycorrhized *M*. *truncatula pt4-2* roots, showing a premature arbuscule degeneration (PAD; **B**: a,b,e,f) or Non-PAD phenotype (**B**: c,d,g,h). GUS stainings were performed for 4–8 hours. Alexa WGA Fluor 488 stainings are shown to visualize fungal colonization. Close-up views of PAD and Non-PAD arbuscules are shown inside red angles.

Similar to the results for *ram1-1* mutants (Fig. 3), the promoters of *MtGras1* and *MtGras7* are inactive in *pt4-2* roots, while the *MtRad1*, *MtGras4*, and *MtGras6* promoters are still functional in root areas containing the typical prematurely degrading, stunted arbuscules that were regularly observed for *pt4-2* mutants^53^ (Fig. 5). On the other hand, since the *pt4-2* stunted arbuscule phenotype was not absolutely stable in our growth conditions, some infection units from *pt4-2* roots, that in other areas showed typical premature arbuscule degeneration, occasionally developed WT-like arbuscules. Interestingly, wild-type like *MtGras1* and *MtGras7* promoter activities were now observed (Fig. 5Ba–h). This local phenomenon suggests that *MtGras1* and *MtGras7* activation is dependent on a particular stage of arbuscule development that, when modulated by endogenous or exogenous conditions, as e.g. demonstrated for N-starvation^53^; can alleviate the *pt4-2* phenotype of premature arbuscule degeneration. It thus appears that *MtGras1* and *MtGras7* activation does not simply depend on the *pt4-2* genotype, but on the existence of a symbiotic interface beneficial for the plant, enabling the development of mature arbuscules.

A summary of *MtGras* activity in *ram1-1* and *pt4-2* mutants is presented in Supplementary Figure S3. In both mutants, *MtGras1* and *MtGras7* expression is hardly detectable by real-time RT-PCR experiments or histological studies of promoter activity, while *MtGras4*, *MtGras6*, and *MtRad1* expression comparable to mycorrhizal wild type roots is observed. With respect to the results from our mutant studies, AM-related GRAS TF genes can thus be divided into two groups, being either MtRAM1- and MtPT4-dependent or -independent.

### MtGRAS4 and MtGRAS7 form a regulatory module within the network of AM-related GRAS TFs

**Figure 6:**
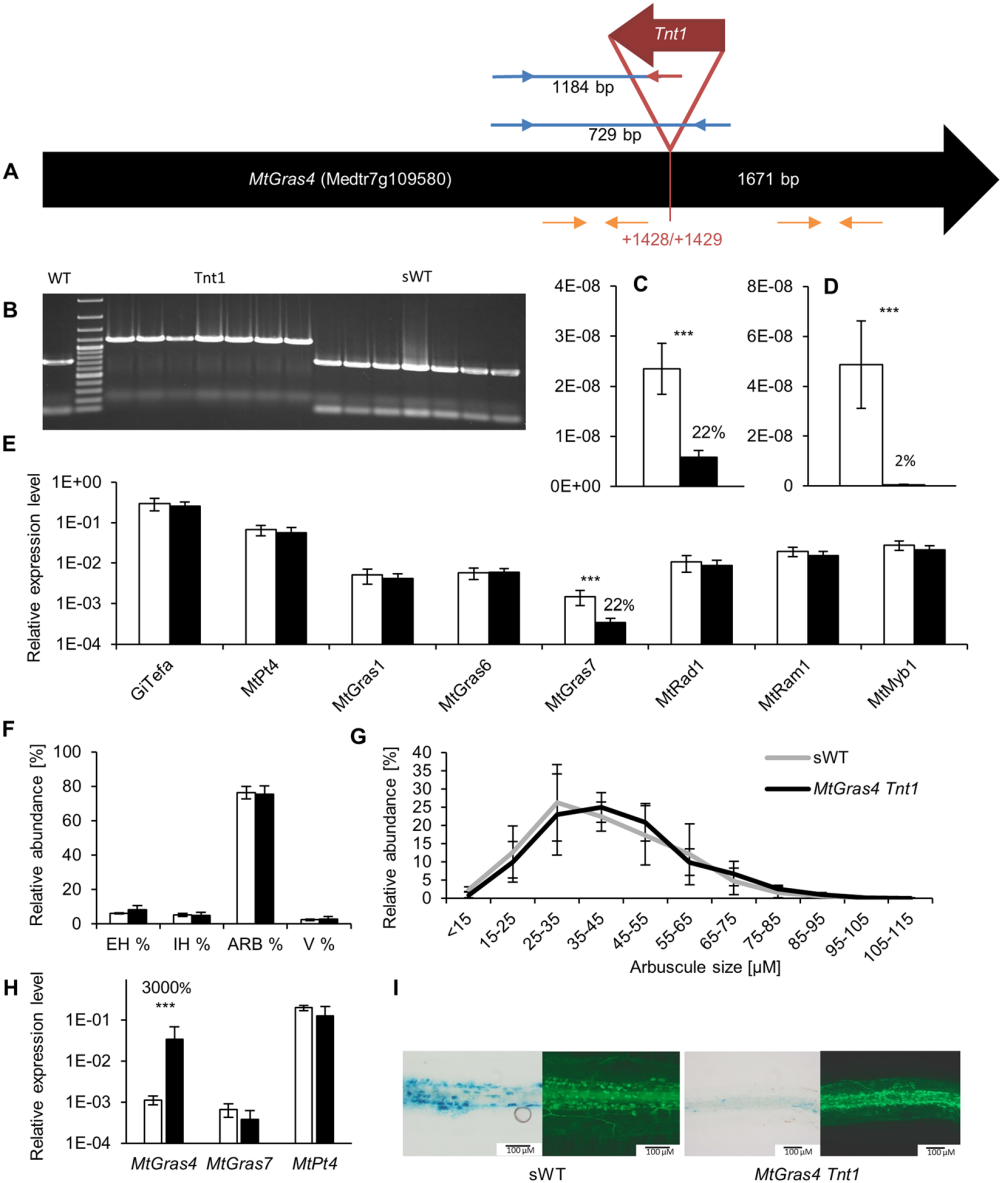
Molecular and phenotypical analysis of the *MtGras4 Tnt1* insertion-carrying line NF4813. (**A**) Schematic illustration of the *Tnt1* insertion site (red), primer position for genomic PCR-amplification (blue and red arrows), and position of real-time RT-PCR primers (orange) in the exon of *MtGras4* (black). (**B**) Leaf disc multiplex PCR identification of homozygous, *MtGras4 Tnt1* insertion-carrying NF4813 (*Tnt1*), *MtGras4*-segregating NF4813 wild type (sWT), and R108 wild type (WT) plants. A 50 bp DNA ladder shown as standard. A full-length gel is presented in Supplementary Fig. S4. (**C**,**D**) Relative amount of *MtGras4* transcript regions located 5′ (**C**) and 3′ (**D**) of the *Tnt1* insertion. (**E**) Relative expression of selected genes in homozygous *MtGras4 Tnt1* mutant (black) and segregating wild type plants (white). Transcript amounts are shown relative to *MtTefα* expression. Roots were harvested at 35 dpi with *R*. *irregularis*. n = 8 biological replicates, error bars represent standard deviations. Numbers indicate the percental expression level compared to control roots. (**F**) Quantification of fungal structures in homozygous, *MtGras4 Tnt1* mutant (black) and segregating wild type plants (white). Roots were harvested at 42 dpi with *R*. *irregularis*. Root systems were grouped into four biological replicates each containing a pool of four roots. Standard deviations are indicated as error bars. EH = External hyphae only; IH = Internal Hyphae only; Arb = arbuscules; V = Vesicles (no arbuscules). (**G**) Distribution of arbuscule sizes in mycorrhized homozygous *MtGras4 Tnt1* mutant and segregating wild type plants. Sizes were measured from 1696 arbuscules in *MtGras4 Tnt1* mutant (black) and 1207 arbuscules in segregating wild type plants (grey). Roots were harvested at 42 dpi with *R*. *irregularis*. Root systems were grouped into four biological replicates each containing a pool of two roots. Standard deviations are indicated as error bars. (**H**) Quantification of *MtGras4*, *MtGras7*, and *MtPt4* in a homozygous *MtGras4 Tnt1* mutant complemented with a −1206/+2086 genomic region of the *M*. *truncatula* A17 *MtGras4* gene (black), and empty vector control roots (white). N = 6 biological replicates, error bars represent standard deviations. Numbers indicate percental expression levels compared to control roots. (**I**) Comparison of pMtGras7-*gusA*int activity in mycorrhizal roots of homozygous *MtGras4 Tnt1* mutant and the corresponding segregating wild type (sWT) plants. GUS stainings were performed for 8 hours. Alexa WGA Fluor 488 stainings visualize AM fungal colonization. ***p < 0.001 (Student’s t-test).

As one representative of an MtRAM1- and MtPT4-independent GRAS TF gene, *MtGras4* was further characterized. To understand its role in AM formation, the *Tnt1* transposon insertion line NF4813 was identified in the *Medicago truncatula* mutant database^54^ (Fig. 6A). Plants were inbred to generate a homozygous knockout line, which was tested for the position of the *Tnt1* insertion using genomic PCR (Fig. 6B). Real-time RT-PCR measurements revealed strongly reduced levels of the *MtGras4* 5′ and virtually no remaining *MtGras4* 3′ transcript region up- or downstream of the *Tnt1* insertion site, respectively (Fig. 6C,D).

Among all tested GRAS TF and AM marker genes, the *MtGras4* knockout line showed a reduced expression of *MtGras7*, both on the transcript level (to 22%, Fig. 6E) and the activity of the promoter (Fig. 6I), whereas transcription of all other AM-related GRAS TF genes was unchanged. On the phenotypical level, both the mycorrhization rate and the arbuscule size distribution are unchanged in *MtGras4* knockout mutants (Fig. 6F,G). Complementation of the *M*. *truncatula* R108-based *Tnt1* line with the *M*. *truncatula* A17 *MtGras4* gene led to a restoration of *MtGras7* expression (Fig. 6H), confirming that *MtGras7*, being strongly upregulated during later stages of mycorrhization (Fig. 1), is a direct or indirect target of MtGRAS4. Since *MtGras4* expression was independent of MtRAM1 and MtPT4, we conclude that the MtGRAS4/MtGRAS7 regulatory module operates parallel to the formation of highly branched, functional arbuscules.

### *MtGras1* knockdown affects the expression of other AM-related GRAS TF genes

As an example for a GRAS TF gene dependent on MtRAM1 and MtPT4, *MtGras1* was functionally studied in transgenic RNAi roots exhibiting an *MtGras1* knockdown, since homozygous knockout lines were not available. Due to the fact that RT-PCR measurements indicated effects of an *MtGras1* knockdown on AM-related gene expression, a global transcriptomics approach was pursued.

**Figure 7:**
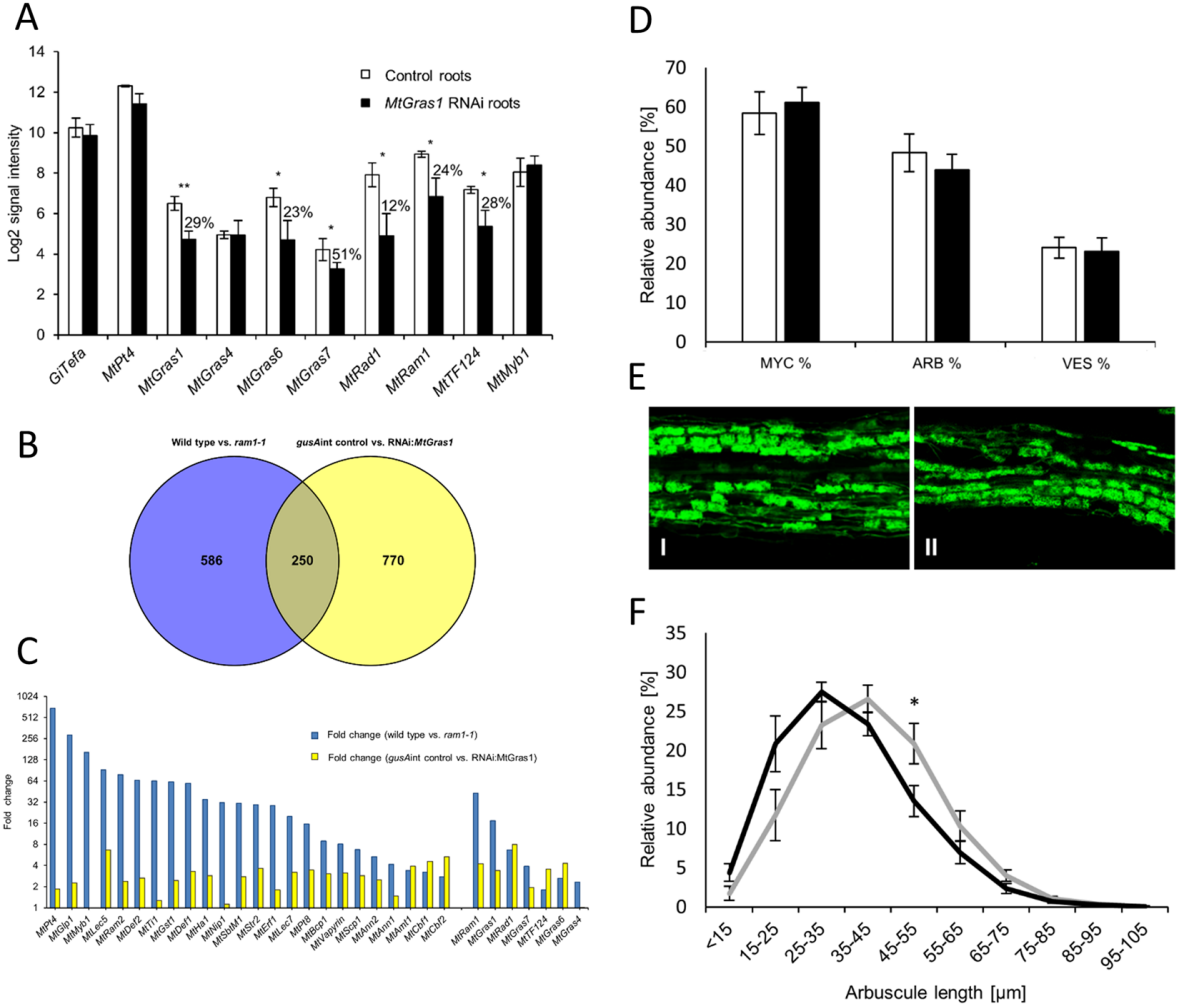
Molecular and phenotypical effects of an *MtGras1* knockdown in mycorrhizal roots. (**A**) Expression of *MtGras1* and selected AM marker genes in transgenic, mycorrhized RNAi:*MtGras1* and RNAi:*gusA*int control roots. Roots were harvested at 35 days post inoculation with *R*. *irregularis*. *Medicago* Transcriptome Assay hybridizations were performed, using three biological replicates per experimental group. The standard error of the mean is shown. Percental values shown are based on the calculated linear signal intensities. Genes shown are defined in the Fig. 2 legend. (**B**) Transcriptional response of *M*. *truncatula ram1-1* and *MtGras1* RNAi roots to colonization with *R*. *irregularis*. Comparison of gene expression in *M*. *truncatula ram1-1* mutant as well as *MtGras1* RNAi roots in relation to control roots. Numbers indicate genes downregulated at least 2-fold (p < 0.05) in the *ram1-1* mutant line or the *MtGras1* RNAi knock-down roots. (**C**) Comparative visualization of fold changes in *ram1-1* (blue) and *MtGras1* RNAi (yellow) roots in comparison to the corresponding control roots. A selection of 31 well-characterized AM-induced marker genes, downregulated at least 2-fold (p < 0.05) in relation to control roots were compared in both mutants. The expression data and identifiers corresponding to the genes studied are listed in Supplementary Table S4. (**D**) Quantification of fungal structures in *MtGras1* RNAi (black) and control roots (white). Roots were harvested at 35 days after inoculation with *R*. *irregularis*. Standard errors are indicated as error bars. MYC, colonized root fragments; ARB, arbuscules; VES, vesicles. (**E**) Alexa WGA Fluor 488 stained mycorrhizal *MtGras1* RNAi (I) and control roots (II). (**F**) Distribution of arbuscule sizes in *MtGras1* RNAi (black) and control roots (grey). Sizes were measured for nine biological replicates of independent *MtGras1* RNAi, and control root systems, respectively (appr. 3000 arbuscules in total for each group). Roots were harvested at 35 days post inoculation with *R*. *irregularis*. Bars represent standard errors. *p < 0.05; **p < 0.01 (Student’s t-test).

A comparative genome-wide gene expression study of *R*. *irregularis*-mycorrhized RNAi:*MtGras1* and RNAi:*gusA*int control roots identified 1020 genes that were at least 2-fold (p < 0.05) downregulated in *MtGras1* knockdown roots, indicating the potential of MtGRAS1 to participate in the regulation of gene expression in AM (Supplementary Table S2). A selection of *MtGras* genes differentially expressed in the *MtGras1* knockdown roots is shown in Fig. 7. In line with initial real-time RT-PCR measurements, the RNA interference construct led to a reduction of *MtGras1* expression to 29% of the wild type level. This 71% *MtGras1* knockdown was able to reduce transcription of the AM-related GRAS TF genes *MtGras6*, *MtGras7*, *MtRad1*^36^, *MtRam1*^33^, and also *MtTF124*^33^, while expression of the fungal marker gene *GiTefa* as well as the *M*. *truncatula MtGras4*, *MtPt4*, and *MtMyb1* genes were not significantly affected (Fig. 7).

Especially the only slight, non-significant (p = 0.11; Fig. 7, Supplementary Table S2) reduction of *MtPt4* expression in *MtGras1* knockdown roots indicates that the formation of active, phosphate-transporting arbuscules is not impaired by a reduced *MtGras1* expression. This was also true for the transcription of the *MtMyb1* gene, encoding a transcription factor controlling arbuscule degeneration^18^, which is not reduced in *MtGras1* knockdown roots (Fig. 7). This indicates that MtGRAS1 does not participate in the initiation of arbuscule degradation, e.g. by activating *MtMyb1*.

Since MtRAM1 was shown to be required for *MtGras1* activation^33^ (Figs 2, 3), it is intriguing that *MtRam1* expression is downregulated to 24% in mycorrhized *MtGras1* knockdown roots (Fig. 7). This finding suggests that similar to the observation for MtRAM1 and MtWRI5a in the regulation of fatty acid biosynthesis^39^, MtRAM1 and MtGRAS1 are part of a regulatory feedback loop that maximizes *MtRam1* transcription and in this case thus might enhance AM-correlated gene expression.

### The transcription profile of *MtGras1* knockdown roots differs substantially from that of *ram1-1* mutants

Facing the large number of genes downregulated in *MtGras1* knockdown roots (Supplementary Table S2), the question arose, to what extent these patterns of transcriptional changes resemble those in an *MtRam1* knockout. To study this, genome-wide expression was recorded in wild type vs. *ram1-1* roots. This experiment revealed that the expression of 836 genes were at least 2-fold (p < 0.05) lower in *ram1-1* knockout roots, including several marker genes for arbuscule function (e.g. *MtPt4*, being transcribed at a 689-fold lower level, Supplementary Tables S3 and S4). Although a limited set of 250 genes is at least 2-fold lower expressed (p < 0.05) in RNAi:*MtGras1* as well as in *ram1-1* roots (Fig. 7B), the major pattern of gene expression regulation is characteristic of either the *MtGras1* knockdown or the *ram1-1* knockout. Specifically, when looking into the detailed pattern of regulation of 31 well-defined AM marker genes (Supplementary Table S4, Fig. 7C), it is evident that most of these are either only (e.g. *MtMyb1*) or much stronger (e.g. *MtPt4*) downregulated in *ram1-1* mutants, indicating that the core gene expression program activated in arbuscule-containing cells is strongly affected in *ram1-1* mutants, but not or only moderately in the *MtGras1* knockdown roots. This finding suggests that although *MtGras1* expression depends on MtRAM1^33^ (Figs 2, 3), MtGRAS1 is not simply a direct target of MtRAM1 to activate downstream genes related to arbuscule formation and function.

### *MtGras1* overexpression does not activate other AM-related GRAS TF genes

The massive transcriptional effects resulting from an *MtGras1* knockdown prompted us to investigate the effect of *MtGras1* overexpression in transgenic *M*. *truncatula* roots. In these experiments, the arbuscule-specific *MtPt4* and the general *ubiquitin3* promoters were used to drive *MtGras1* expression in mycorrhized and non-mycorrhized roots, respectively. Although a 7.4- and 1523-fold *MtGras1*-overexpression was achieved, respectively, leading to comparable amounts of *MtGras1* transcripts in mycorrhized and uninoculated roots (Supplementary Fig. S5), no activation of other GRAS TF or AM marker genes such as *MtPt4* was detected (Supplementary Fig. S5), suggesting that MtGRAS1 is not at the terminal position of a regulatory cascade or requires co-expressed interaction partners to activate transcription of target genes.

### *MtGras1* knockdown roots show a trend towards the development of smaller arbuscules

To address the question, whether an *MtGras1* knockdown influences the fungal colonization of roots or the maturation of arbuscules, phenotypical studies were performed in comparison to control roots.

While no changes in the mycorrhization rate or the frequencies of arbuscules and vesicles were observed (Fig. 7D) and the arbuscules in *MtGras1* knockdown roots did not show symptoms of premature degeneration (Fig. 7E), arbuscule length measurements of mycorrhizal RNAi:*MtGras1* in comparison to RNAi:*gusA*int control roots indicated a shift in the distribution of arbuscule sizes (Fig. 7F). Specifically, *MtGras1* knockdown roots tend to contain a higher proportion of smaller and a lower proportion of large arbuscules, suggesting a delayed or less sustained arbuscule development. In line with the fact that the expression of most marker genes for arbuscule formation and function is not markedly affected in mycorrhized *MtGras1* knockdown roots (Fig. 7B–C), this effect nevertheless appears subtle and is probably part of a fine-tuning of the arbuscule life-cycle.

## Discussion

Colonization of roots by AM fungi ultimately leads to the formation of intracellular arbuscules, functioning as a nutrient exchange interface between plant cells and fungal hyphae. Arbuscule development requires a fundamental transcriptional reprogramming of root cortical cells^44–49,55^, being governed by a suite of AM-activated regulators, including several GRAS TFs^29,33,34,36,40,42,47,55^.

**Figure 8:**
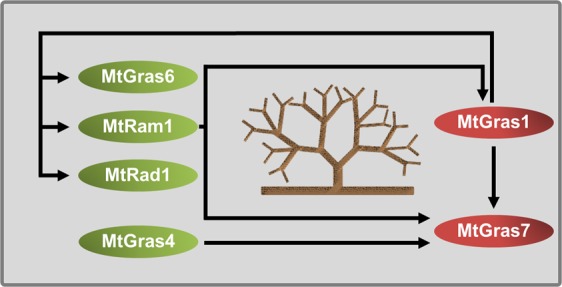
Model of the regulatory circuit of AM-related GRAS TFs revolving arbuscule development. Components of the network are divided into MtRAM1- and MtPT4-independet (MtGRAS4, MtGRAS6, and MtRAD1) as well as -dependent (MtGRAS1, MtGRAS7) GRAS TFs. Black arrows visualize direct or indirect transcriptional activation, including putative and so far unknown mediators.

We show here that the five AM-activated GRAS TF genes *MtGras1*, *MtGras4*, *MtGras6*, *MtGras7*, and *MtRad1*^42^ can be classified based on their dependency on MtRAM1^33^, a key transcription factor controlling arbuscule branching, and MtPT4^9,50^, the major AM-specific phosphate transporter. While *MtGras4*, *MtGras6*, and *MtRad1* are still expressed in the absence of *MtRam1* and *MtPt4*, *MtGras1* and *MtGras7* transcription is abolished in the *ram1-1* and *pt4-2* mutants (Figs 2–5). In our growth conditions, *pt4-2* mutants occasionally developed not only prematurely decaying but also apparently vital arbuscules (Fig. 5Ba–h). This phenomenon demonstrates the dependency of *MtGras1* and *MtGras7* transcription not on the genotype, but on the presence of fully developed arbuscules. Obviously, prematurely decaying arbuscules do not reach the phase of *MtGras1* and *MtGras7* expression, while fully developed arbuscules do. This indicates that with respect to transcriptional control, MtGRAS1 and MtGRAS7 are placed downstream of MtRAM1 action and might thus be related to stages of arbuscule formation, when arbuscule maturation already occurred. In contrast, *MtGras4*, *MtGras6*, and *MtRad1* expression is not abolished in *ram1-1* and *pt4-2* mutants (Supplementary Fig. S3), allowing to conclude that the encoded GRAS TFs are not related to arbuscule maturation and are thus likely connected to developmental stages before arbuscule maturation takes place, although a function independent of MtRAM1 and MtPT4 cannot be ruled out. These findings were visualized in a model, where the AM-related GRAS TFs are positioned relative to the establishment of mature arbuscules (Fig. 8).

To study the function of an MtRAM1- and MtPT4-independent GRAS TF gene, an *MtGras4* insertion mutant in *M*. *truncatula* R108 was analyzed. Although the *MtGras4* knockout downregulates *MtGras7*, which in contrast to MtGRAS4 is MtRAM1- and MtPT4-dependent, it does not affect AM fungal colonization or the arbuscule size distribution (Fig. 6). The *MtGras4* knockout, inducing a reduced *MtGras7* transcription in the *M*. *truncatula* R108 *MtGras4* mutants, was successfully complemented by expressing an *M*. *truncatula* A17 *MtGras4* gene, indicating a conserved regulation in *M*. *truncatula* R108 and Jemalong A17 regarding these genes. Interestingly, analyses of *MtGras7* and *MtGras4* upregulation during the AM time course revealed different patterns (Fig. 1). While *MtGras7* appeared to be expressed in an arbuscule-correlated manner that follows *MtPt4* activation, *MtGras4* expression was already detectable from the onset of fungal colonization. In line with the MtRAM1- and MtPT4-dependency of *MtGras7* activation (Figs 2, 3), this further indicates that MtGRAS7 functions in the later stages of arbuscule development, when maturation already took place. In this respect, the dependency of *MtGras7* on the MtRAM1- and MtPT4-independent GRAS TF MtGRAS4 is even more remarkable, since it demonstrated a connection between more early and MtRAM1-independent (*MtGras4*) and more later, MtRAM1-dependent stages (*MtGras7*) of arbuscule formation. The somewhat outstanding position of the MtGRAS4/MtGRAS7 module within the GRAS TF network is also reflected on the sequence level, where MtGRAS4 and MtGRAS7 share a common clade, being isolated from the rest of the GRAS TFs investigated (Supplementary Fig. S2).

In spite of its dependency on MtRAM1 and MtPT4 (Supplementary Fig. S3), MtGRAS1 was also found to regulate components placed more upstream of arbuscule development, namely *MtGras6*, *MtRad1*, and remarkably also *MtRam1* (Fig. 7A). In addition, the AM-related GRAS TF gene and *MtRam1*-homologue *MtTf124*^33^ appears downregulated in *MtGras1* knockdown roots (Supplementary Table S4). Due to the fact that MtGRAS1 regulates *MtRam1* expression (Fig. 7A), while MtRAM1 on the other hand is required for *MtGras1* activation (Figs 2 and 3), we propose the existence of a regulatory feedback loop, where MtGRAS1 enhances *MtRam1* transcription, thus stimulating its own activation as well as the expression of MtRAM1-regulated target genes, ultimately leading to the establishment of a functional, highly branched arbuscule (Fig. 8). Since it was demonstrated that MtRAM1 is not able to directly stimulate *MtGras1* expression^33^, the activation of *MtGras1* thus has to be dependent on additional, MtRAM1-dependent regulators. It is likely that currently unknown mediators also have to be assumed for most of the relationships shown in the regulatory network visualized in Fig. 8.

Interestingly, *MtGras1* expression was downregulated in *pt4-2* mutants colonized by *Rhizophagus irregularis* (Figs 4, 5) and in *pt4-1* mutants mycorrhized with *Gigaspora gigantea*^18^, indicating that the encoded GRAS TF acts downstream of the formation of functional arbuscules. It is thus tempting to hypothesize that MtGRAS1 acts as a checkpoint at a certain point of the later stages of the arbuscule life cycle, where the initial signaling for arbuscule development and branching via MtRAM1 is completed, and the morphological expansion of the functional, phosphate-transporting fungal interface needs to be accelerated or sustained. The slight shift towards the formation of smaller arbuscules observed in *MtGras1* RNAi roots (Fig. 7D–F) might thus be caused by the lack of MtGRAS1-activated *MtRam1* transcription (Fig. 7A), which would lead to a slower or less sustained, MtRAM1-controled, arbuscule branching. The effect on the arbuscule size distribution is nevertheless small and possibly also suffers from the non-synchronized mycorrhization process, which masks subtle differences in arbuscules sizes.

On the protein level, MtGRAS1 was shown to interact with MtRAM1 in yeast two-hybrid assays^33^, which indicates a joint function of these transcription factors. However, we were able to show that an RNAi-mediated knockdown of *MtGras1* leads to a different molecular phenotype than a knockout of *MtRam1*. Although there are overlaps in the genes regulated by MtGRAS1 and MtRAM1, *ram1-1* mutants show a much stronger downregulation of AM-induced genes characteristic of the presence of functional arbuscules and a strong effect on arbuscule branching^33^, which is not the case for *MtGras1* RNAi roots (Fig. 7A). The different transcription pattern of mycorrhized *MtGras1* RNAi roots and mycorrhized *ram1-1* mutants is thus in line with a modifying function of MtGRAS1 during the establishment of a functional arbuscule interface. Although these comparative transcription patterns (Fig. 7B) were derived from studies of knockout mutants (*MtRam1*) and transgenic knockdown roots (*MtGras1*), the fundamentally different expression pattern observed in *ram1-1* and *MtGras1* RNAi roots most likely excludes that MtGRAS1 itself acts as a key independent regulator of arbuscule formation, downstream of MtRAM1. An additional hint that MtGRAS1 solely is unable to activate AM-related genes derives from our overexpression experiments, where an upregulated *MtGras1* transcription had no effect on selected target genes, probably since MtGRAS1 requires an interaction partner such as MtRAM1^33^.

In addition to the more upstream components regulating arbuscule formation, MtGRAS1 also regulates *MtGras7* (Fig. 7A), which is also MtRAM1- and MtPT4-dependent and is thus located at a similar position relative to the formation of mature arbuscules. Incidentally, the downregulation of *MtGras7* in *MtGras1* knockdown roots might explain why *MtGras7* expression is reduced but still detectable in the *MtGras4* insertion mutant (that still expresses *MtGras1*, Fig. 6).

The effect of MtGRAS1 on *MtGras7* transcription implies that *MtGras7* can be activated by both the AM-related MtGRAS4 and the AM-specific MtGRAS1 TF in an independent manner, suggesting that MtRAM1-/MtPT4-dependent (MtGRAS1 and its targets including *MtGras7*) and -independent regulatory modules (MtGRAS4 and its target *MtGras7*) are connected. The complex network of AM-related and AM-specific GRAS TFs might thus contribute to the balanced expression of AM-related genes at the arbuscule interface, where different physiological and nutritional conditions have to be integrated.

## Conclusions

Relying on gene expression and histological studies in symbiotic mutants defective in arbuscule branching or in the formation of phosphate-transporting arbuscules as well as on functional analyses in transgenic knockdown roots or knockout lines, we provide evidence that the network of AM-activated *M*. *truncatula* GRAS TFs consists of interconnected modules, including an MtRAM1-MtGRAS1 regulatory feedback loop. *MtGras1* knockdown roots displayed normal colonization by AM fungi, but a trend towards the formation of smaller arbuscules was apparent. Although mutants in the AM-specific GRAS TFs RAM1 and RAD1 display more severe AM-related phenotypes in *M*. *truncatula* and *L*. *japonicus*^33,34,36,42^, our investigation on additional AM-activated *MtGras* genes provides evidence that a regulatory circuit of multiple GRAS TFs, showing differential dependencies on arbuscule branching and the formation of phosphate-transporting arbuscules, governs and sustains arbuscule development. We propose that this regulatory circuit allows a flexible response of the symbiotic interface towards the external (e. g. nutrient availability^50,53^; and internal (e. g. plant hormones^20,41^) stimuli that together influence and adapt the arbuscule life cycle under changing conditions.

## Methods

### Cloning of promoter-gusAint fusions and histological analyses

Promoter sequences of *MtGras* genes were amplified from genomic DNA of *M*. *truncatula* using oligonucleotides specified in Supplementary Table S5. PCR-fragments were cloned into pGUS-INT^56^, in front of the *gusA*int reporter gene cassette. The resulting transcriptional fusions were released using *Spe*I and subcloned into the *Sma*I-digested binary vector pRedRoot^57^, after fill-in of 5′ overhangs using the Klenow fragment.

GUS assays were performed by incubating roots in GUS staining buffer^56^ for 4 to 8 hours at 37 °C, if not stated differently. Counterstaining of fungal material was performed using Alexa WGA Fluor 488 (Thermo Fisher Scientific, Langenselbold, Germany), as described above.

### Cloning of knock-down and overexpression constructs

An RNAi construct for *MtGras1* was generated by amplification and recombination of a 379 bp long fragment of the *MtGras1* coding region into pDONR^TM^221 (Gateway®-System, Invitrogen, Karlsruhe, Germany) via the BP-, and subsequently into the binary vector pK7GWIWG2(II)-*Q10*:DsRED^58^ via the LR-reaction. Specificity of the *MtGras1* RNAi target sequence was verified via BLAST searches against the current release of the *M*. *truncatula* genome^51^ and by using the siFi software that predicts potential off-targets^59^.

*MtGras1* overexpression constructs were generated by PCR-amplification and cloning of the *MtGras1* coding sequence into the vectors 315p9RFP-Pt4-Expr and 917p9RFP-ubi3-Expr^15^, containing either the *M*. *truncatula MtPt4*^9^ or the *A*. *thaliana ubiquitin 3* promoter (*AtUbq3*^60^), respectively.

### Plant growth, inoculation with AM fungi and staining of fungal structures

*Medicago truncatula* Gaertn cv Jemalong genotype A17 seeds were surface-sterilized and scarified as reported^61^. Plants were grown in the climate chamber (relative humidity: 60%; photosynthetic photon flux: 150 μmol m^−2^ s^−1^), using a 16 h light (22 °C) and 8 h dark (18 °C) regime. *M*. *truncatula* R108 plants harbouring a *Tnt1* insertion and corresponding control plants were surface-sterilized and scarified as described above and grown in a phytocabinet (Klimaschrank KPS 1700 Weisshaar, Bad Salzuflen, Germany), using a 16 h light (22 °C) and 8 h dark (22 °C) regime (Osram FLUORA neon tubes, Osram, München, Germany; photosynthetic photon flux: 100 μmol m^−2^ s^−1^) and a relative humidity of 60%.

Transgenic roots were obtained by *Agrobacterium rhizogenes* ARqua1 mediated transformation of *M*. *truncatula* seedlings according to^62^. Bacteria were grown for two days at 30 °C on selective TY (0.5 g/l tryptone; 0.3 g/l yeast extract; 0.07 g/l CaCl_2_ × 2H_2_O) agar plates. Cells were resuspended in 10 ml PS buffer (40 mM Na_2_HPO_4_ × 2H_2_O, 85 mM NaCl, 17 mM KH_2_PO_4_; pH 7). The agrobacterium solution was injected into the hypocotyl using a syringe. Seedlings were planted into sterile Seramis® (Seramis GmbH, Mogendorf, Germany), incubated at 18 °C in the dark for 16 hours and were then transferred into a phytochamber. To detect transgenic roots, plants were screened after 4 weeks for dsRed expression using a stereomicroscope (Leica MZ 10 F, Leica Microsystems, Wetzlar, Germany).

After four weeks of growth (or four weeks after induction of transgenic roots), each plantlet or composite plant was mycorrhized by inoculation in a small amount of water with 2000 spores of germinating *Glomus intraradices* Schenck and Smith DAOM197198 spores (Premier Tech Biotechnologies, Rivière-de-Loup, Québec, Canada), having been reassigned to *Rhizophagus irregularis* (Błaszk., Wubet, Renker, and Buscot) C. Walker & A. Schüßler comb. nov.^63^. After 3–4 hours of inoculation, plantlets were potted into 8 × 7 × 7 cm (height × width × depth) pots filled with sterile Seramis® (Seramis GmbH, Mogendorf, Germany). Each pot contained two plants, and remaining spore solution was directly pipetted onto the root surface while potting. Mycorrhizal and non-mycorrhizal plants were fertilized with half-strength Hoagland’s solution^64^ containing 20 µM phosphate. The solution was prepared with deionized water, pH was adjusted to 6.4 with KOH.

To visualize fungal colonization, roots were incubated in 10% (w/v) KOH at 95 °C for 7 min, repeatedly rinsed with water and incubated in 1x PBS buffer (0.14 M NaCl, 2.7 mM KCl, 1 mM Na_2_HPO_4_ × 2H_2_O, 1.8 mM KH_2_PO_4_; pH 7.3) containing 20 µg/ml Alexa WGA Fluor™ 488 (Thermo Fisher Scientific, Langenselbold, Germany) conjugate overnight. Photo documentation was performed using a Leica MZ 10 F stereomicroscope (Leica Microsystems, Wetzlar, Germany) equipped with an Olympus XC50 camera (Olympus, Hamburg, Germany), a Zeiss Axio Observer Z1 microscope equipped with an AxioCam ICc1 (Carl Zeiss AG, Oberkochen, Germany), and a confocal microscope (Leica TCS SP8 MP, Sohns, Germany). Quantification of fungal colonization was performed using the gridline intersection method^65^. For arbuscule size determination, confocal images of arbuscules were analyzed using the Fiji software^66^. Nine independent roots or four independent pools of two individual roots each were used to determine arbuscule sizes, based on the procedure described by^50,53^ and^18^.

To study gene expression in transgenic *M*. *truncatula* A17 roots expressing an *MtGras1* RNAi construct (RNAi:*MtGras1* roots) in comparison to a *gusA*int gene (RNAi:*gusA*int control roots), composite plants were mycorrhized with *R*. *irregularis* spores as described above and harvested after 54 dpi. To analyse gene expression in *M*. *truncatula ram1-1* roots in comparison to *M*. *truncatula* A17 control roots, roots were mycorrhized with *R*. *irregularis* spores as described above and harvested after 35 dpi. In all experiments, harvesting time points were selected depending on the mycorrhization rate in the different mutants, in order to obtain sufficient and comparable colonization levels.

### Analysis of the *Tnt1* insertion line NF4813

The *Tnt1*^54^ insertion line NF4813 (based on *M*. *truncatula* R108), harbouring a *Tnt1* insertion in the exon of *MtGras4* after position +1428, was obtained from the Noble Research Institute (Ardmore, Oklahoma, USA). No stable, homozygous *Tnt1* lines could be obtained for other candidate genes. Plants were screened for the *Tnt1* insertion via direct PCR from leaf discs using the Phire Plant Direct PCR Kit (Thermo Fisher Scientific, Langenselbold, Germany). The PCR was performed using a *Tnt1* binding (*Tnt1*-F) and two gene-specific primers (NF4813_16_for, NF4813_16_rev). Homozygous plants were selected, selfed, and used for seed propagation. Segregating plants being wild type with respect to the *MtGras4* locus were propagated to obtain control plants.

### RNA isolation and real-time RT-PCR

RNA was isolated using the RNeasy Plant Mini Kit (Qiagen, Hilden, Germany). Tissue disruption was carried out via FastPrep®−24 (MP Biomedicals, Santa Ana, USA). Real-time RT-PCR analyses were performed using the SensiFAST™ SYBR® No-ROX One-Step Kit (Qiagen, Hilden, Germany), using primers listed in Supplementary Table S6. Primers were tested for specificity before use. 5 ng of total RNA were used as a template in a 20 µl reaction. RT-PCR reactions followed a three-step cycling program: Reverse transcription at 45 °C for 10 min; polymerase activation at 95 °C for 2 min; PCR amplification with 40 cycles at 95 °C for 5 sec, 55 °C for 10 sec, and 72 °C for 8 sec. The housekeeping gene *MtTefa* (Medtr6g021805.1 in the *M*. *truncatula* genome^51^) encoding a translation elongation factor was used for normalization. Each biological replicate was measured in three technical replicates. Average values were used to calculate gene expression levels via the 2^−ΔCT^ method with ΔCT = CT_gene_ − CT_*MtTefa*_. Statistical significances were calculated using a two-tailed Student’s t test in MS Excel 2016 (Microsoft Corp., Redmond, Washington, USA).

### Hybridization and data evaluation of GeneChip® *Medicago* Transcriptome Assays

Biotinylated aRNA obtained from 100 ng of total RNA for each sample was fragmented as recommended (GeneChip® Medicago Transcriptome Assay, ThermoFisher Scientific, Schwerte, Germany). The size distribution of the fragmented aRNA was assessed via an Agilent bioanalyzer (Agilent Technologies, Böblingen, Germany) using an RNA 6000 assay. Standard hybridization, post-hybridization wash and double-staining as well as scanning was done as specified for GeneChip® Medicago Transcriptome Assays (ThermoFisher Scientific, Schwerte, Germany).

Cel files were analyzed using the Expression Console and Transcriptome Analysis Console software (both ThermoFisher Scientific, Schwerte, Germany). Normalization was performed via the Robust Multichip Average algorithm, intensity values for each probe set were log2-transformed and averaged across the three biological replicates using the Tukey’s Bi-weight average algorithm, and expression ratios were evaluated statistically via tools of the Transcriptome Analysis Console (ThermoFisher Scientific, Schwerte, Germany).

Original annotations of the genes represented on the GeneChip® *Medicago* Transcriptome Assays were updated by annotations from the *M*. *truncatula* genome version 4.0^51^, and mapped to probe sets from the GeneChip *Medicago* genome arrays that were used to construct the *Medicago* Gene Expression Atlas^52^ as well as to UniProt^67^. Venn diagrams were drawn using the VENNY software^68^.

## Supplementary information

Supplentary Information

Supplementary Dataset S2

Supplementary Dataset S3

## Data Availability

All data generated or analysed during this study are included in this published article and its supplementary information files. In addition, GeneChip® *Medicago* Transcriptome Assay data are deposited in the Gene Expression Omnibus repository (https://www.ncbi.nlm.nih.gov/geo/, accession number GSE108867).

## References

[1] Schüssler A, Schwarzott D, Walker C (2001). A new fungal phylum, the Glomeromycota: phylogeny and evolution. Mycological Research.

[2] Smith, S. E. & Read, D. J. *Mycorrhizal Symbiosis*. (Academic Press, 1997).

[3] Smith SE, Smith FA (2011). Roles of arbuscular mycorrhizas in plant nutrition and growth: new paradigms from cellular to ecosystem scales. Annual Review of Plant Biology.

[4] Genre A, Chabaud M, Timmers T, Bonfante P, Barker DG (2005). Arbuscular mycorrhizal fungi elicit a novel intracellular apparatus in Medicago truncatula root epidermal cells before infection. Plant Cell.

[5] Parniske M (2008). Arbuscular mycorrhiza: the mother of plant root endosymbioses. Nature Reviews Microbiology.

[6] Harrison MJ (1999). Molecular and cellular aspects of the arbuscular mycorrhizal symbiosis. Annual Review of Plant Biology.

[7] Pumplin N (2010). Medicago truncatula Vapyrin is a novel protein required for arbuscular mycorrhizal symbiosis. Plant Journal.

[8] Cox G, Tinker P (1976). Translocation and Transfer of Nutrients in Vesicular-Arbuscular Mycorrhizas. I. The Arbuscule and Phosphorus Transfer: A Quantitative Ultrastructural Study. New Phytologist.

[9] Harrison MJ, Dewbre GR, Liu J (2002). A phosphate transporter from Medicago truncatula involved in the acquisition of phosphate released by arbuscular mycorrhizal fungi. Plant Cell.

[10] Bonfante P, Genre A (2010). Mechanisms underlying beneficial plant-fungus interactions in mycorrhizal symbiosis. Nature Communications.

[11] Baier MC (2010). Knockdown of the symbiotic sucrose synthase MtSucS1 affects arbuscule maturation and maintenance in mycorrhizal roots of Medicago truncatula. Plant Physiology.

[12] Garcia K, Doidy J, Zimmermann SD, Wipf D, Courty PE (2016). Take a trip through the plant and fungal transportome of mycorrhiza. Trends in Plant Science.

[13] Lanfranco, L., Bonfante, P. & Genre, A. The Mutualistic Interaction between Plants and Arbuscular Mycorrhizal Fungi. Microbiol Spectrum **4**, FUNK-0012-2016 (2016). 10.1128/microbiolspec.FUNK-0012-2016 28087942

[14] Harrison MJ (2005). Signaling in the arbuscular mycorrhizal symbiosis. Annual Review of Microbiology.

[15] Devers EA, Teply J, Reinert A, Gaude N, Krajinski F (2013). An endogenous artificial microRNA system for unraveling the function of root endosymbioses related genes in Medicago truncatula. BMC Plant Biology.

[16] Luginbuehl LH, Oldroyd GED (2017). Understanding the Arbuscule at the Heart of Endomycorrhizal Symbioses in Plants. Current Biology.

[17] Gutjahr C, Parniske M (2017). Cell Biology: Control of Partner Lifetime in a Plant-Fungus Relationship. Current Biology.

[18] Floss DS (2017). A Transcriptional Program for Arbuscule Degeneration during AM Symbiosis Is Regulated by MYB1. Current Biology.

[19] Pimprikar P, Gutjahr C (2018). Transcriptional Regulation of Arbuscular Mycorrhiza Development. Plant and Cell Physiology.

[20] Floss DS, Levy JG, Levesque-Tremblay V, Pumplin N, Harrison MJ (2013). DELLA proteins regulate arbuscule formation in arbuscular mycorrhizal symbiosis. Proceedings of the National Academy of Sciences.

[21] Uhe M (2018). The mycorrhiza-dependent defensin MtDefMd1 of Medicago truncatula acts during the late restructuring stages of arbuscule-containing cells. PLoS ONE.

[22] Riechmann JL (2000). Arabidopsis transcription factors: genome-wide comparative analysis among eukaryotes. Science.

[23] Shiu S-H, Shih M-C, Li W-H (2005). Transcription factor families have much higher expansion rates in plants than in animals. Plant Physiology.

[24] Peng J (1997). The Arabidopsis GAI gene defines a signaling pathway that negatively regulates gibberellin responses. Genes and Development.

[25] Silverstone AL, Ciampaglio CN, Sun T (1998). The Arabidopsis RGA gene encodes a transcriptional regulator repressing the gibberellin signal transduction pathway. Plant Cell.

[26] Di Laurenzio L (1996). The SCARECROW gene regulates an asymmetric cell division that is essential for generating the radial organization of the Arabidopsis root. Cell.

[27] Zhang H (2017). Genome-wide characterization of GRAS family genes in Medicago truncatula reveals their evolutionary dynamics and functional diversification. PLoS ONE.

[28] Song L, Tao L, Cui H, Ling L, Guo C (2010). Genome-wide identification and expression analysis of the GRAS family proteins in Medicago truncatula. Acta Physiologiae Plantarum.

[29] Bucher M, Hause B, Krajinski F, Küster H (2014). Through the doors of perception to function in arbuscular mycorrhizal symbioses. New Phytologist.

[30] Hirsch S (2009). GRAS proteins form a DNA binding complex to induce gene expression during nodulation signaling in Medicago truncatula. Plant Cell.

[31] Gobbato E (2012). A GRAS-type transcription factor with a specific function in mycorrhizal signaling. Current Biology.

[32] Gobbato E (2013). RAM1 and RAM2 function and expression during arbuscular mycorrhizal symbiosis and Aphanomyces euteiches colonization. Plant Signaling and Behavior.

[33] Park H-J, Floss DS, Levesque-Tremblay V, Bravo A, Harrison MJ (2015). Hyphal Branching during Arbuscule Development Requires Reduced Arbuscular Mycorrhiza1. Plant Physiology.

[34] Pimprikar P (2016). A CCaMK-CYCLOPS-DELLA Complex Activates Transcription of RAM1 to Regulate Arbuscule Branching. Current Biology.

[35] Rich MK (2015). The Petunia GRAS Transcription Factor ATA/RAM1 Regulates Symbiotic Gene Expression and Fungal Morphogenesis in Arbuscular Mycorrhiza. Plant Physiology.

[36] Xue L (2015). Network of GRAS transcription factors involved in the control of arbuscule development in Lotus japonicus. Plant Physiology.

[37] Hohnjec N, Czaja-Hasse LF, Hogekamp C, Küster H (2015). Pre-announcement of symbiotic guests: transcriptional reprogramming by mycorrhizal lipochitooligosaccharides shows a strict co-dependency on the GRAS transcription factors NSP1 and RAM1. BMC Genomics.

[38] Bravo A, Brands M, Wewer V, Dörmann P, Harrison MJ (2017). Arbuscular mycorrhiza-specific enzymes FatM and RAM2 fine-tune lipid biosynthesis to promote development of arbuscular mycorrhiza. New Phytologist.

[39] Jiang Y (2018). Medicago AP2-domain transcription factor WRI5a is a master regulator of lipid biosynthesis and transfer during mycorrhizal symbiosis. Molecular Plant.

[40] Xue L (2018). AP2 transcription factor CBX1 with a specific function in symbiotic exchange of nutrients in mycorrhizal Lotus japonicus. Proceedings of the National Academy of Sciences of the United States of America.

[41] Foo, E., Ross, J. J., Jones, W. T. & Reid, J. B. Plant hormones in arbuscular mycorrhizal symbioses: an emerging role for gibberellins. *Annals of Botany* **111**, 769–779. 10.1093/aob/mct041 PMC3631329 23508650

[42] Rey T (2017). The Medicago truncatula GRAS protein RAD1 supports arbuscular mycorrhiza symbiosis and Phytophtora palmivora susceptibility. Journal of Experimental Botany.

[43] Heck C (2016). Symbiotic fungi control plant root cortex development through the novel GRAS transcription factor MIG1. Current Biology.

[44] Hohnjec N, Vieweg MF, Pühler A, Becker A, Küster H (2005). Overlaps in the transcriptional profiles of Medicago truncatula roots inoculated with two different Glomus fungi provide insights into the genetic program activated during arbuscular mycorrhiza. Plant Physiology.

[45] Küster H (2007). Identification and expression regulation of symbiotically activated legume genes. Phytochemistry.

[46] Gomez SK (2009). Medicago truncatula and Glomus intraradices gene expression in cortical cells harboring arbuscules in the arbuscular mycorrhizal symbiosis. BMC Plant Biology.

[47] Hogekamp C (2011). Laser microdissection unravels cell-type-specific transcription in arbuscular mycorrhizal roots, including CAAT-box transcription factor gene expression correlating with fungal contact and spread. Plant Physiology.

[48] Gaude N, Bortfeld S, Duensing N, Lohse M, Krajinski F (2012). Arbuscule-containing and non-colonized cortical cells of mycorrhizal roots undergo extensive and specific reprogramming during arbuscular mycorrhizal development. Plant Journal.

[49] Hogekamp C, Küster H (2013). A roadmap of cell-type specific gene expression during sequential stages of the arbuscular mycorrhiza symbiosis. BMC Genomics.

[50] Javot H, Penmetsa RV, Terzaghi N, Cook DR, Harrison MJ (2007). A Medicago truncatula phosphate transporter indispensable for the arbuscular mycorrhizal symbiosis. Proceedings of the National Academy of Sciences of the United States of America.

[51] Tang H (2014). An improved genome release (version Mt4.0) for the model legume Medicago truncatula. BMC Genomics.

[52] Benedito VA (2008). A gene expression atlas of the model legume Medicago truncatula. Plant Journal.

[53] Javot H (2011). Medicago truncatula mtpt4 mutants reveal a role for nitrogen in the regulation of arbuscule degeneration in arbuscular mycorrhizal symbiosis. Plant Journal.

[54] Tadege M (2008). Large scale insertional mutagenesis using Tnt1 retrotransposon in the model legume Medicago truncatula. Plant Journal.

[55] Guether M (2009). Genome-wide reprogramming of regulatory networks, transport, cell wall and membrane biogenesis during arbuscular mycorrhizal symbiosis in Lotus japonicus. New Phytologist.

[56] Küster H, Quandt HJ, Broer I, Perlick AM, Pühler A (1995). The promoter of the Vicia faba L. VfENOD-GRP3 gene encoding a glycine-rich early nodulin mediates a predominant gene expression in the interzone II-III region of transgenic Vicia hirsuta root nodules. Plant Molecular Biology.

[57] Limpens E (2004). RNA interference in Agrobacterium rhizogenes-transformed roots of Arabidopsis and Medicago truncatula. Journal of Experimental Botany.

[58] Limpens E (2005). Formation of organelle-like N2-fixing symbiosomes in legume root nodules is controlled by DMI2. Proceedings of the National Academy of Sciences of the United States of America.

[59] Lueck, S. siFi_ Software for long double-stranded RNAi-target design and off-target prediction, 10.5447/IPK/2017/9 (2017).

[60] Norris SR, Meyer SE, Callis J (1993). The intron of Arabidopsis thaliana polyubiquitin genes is conserved in location and is a quantitative determinant of chimeric gene expression. Plant Molecular Biology.

[61] Hohnjec N, Perlick AM, Pühler A, Küster H (2003). The Medicago truncatula sucrose synthase gene MtSucS1 is activated both in the infected region of root nodules and in the cortex of roots colonized by arbuscular mycorrhizal fungi. Molecular Plant-Microbe Interactions.

[62] Vieweg MF (2004). The promoter of the Vicia faba L. leghemoglobin gene VfLb29 is specifically activated in the infected cells of root nodules and in the arbuscule-containing cells of mycorrhizal roots from different legume and nonlegume plants. Molecular Plant-Microbe Interactions.

[63] Stockinger H (2014). The Largest Subunit of RNA Polymerase II as a New Marker Gene to Study Assemblages of Arbuscular Mycorrhizal Fungi in the Field. PLoS ONE.

[64] Arnon DI, Hoagland DR (1940). Crop production in artificial culture solutions and in soils with special reference to factors influencing yields and absorption of inorganic nutrients. Soil Science.

[65] Brundrett, M., Bougher, N., Dell, B., Grove, T. & Malajczuk, N. Working with Mycorrhizas in Forestry and Agriculture. *Canberra: Australian Centre for International Agricultural Research* (1996).

[66] Schindelin J (2012). Fiji: an open-source platform for biological-image analysis. Nat Methods.

[67] UniProt Consortium T (2018). UniProt: the universal protein knowledgebase. Nucleic Acids Research.

[68] Oliveros, J. C. Venny. An interactive tool for comparing lists with Venn diagrams, http://bioinfogp.cnb.csic.es/tools/venny/index.html.

[69] Sokolski S (2010). Conspecificity of DAOM 197198, the model arbuscular mycorrhizal fungus, with Glomus irregulare: molecular evidence with three protein-encoding genes. Botany.

[70] Liu J (2007). Arbuscular mycorrhizal symbiosis is accompanied by local and systemic alterations in gene expression and an increase in disease resistance in the shoots. Plant Journal.

