# Supplementary material for: Insights into the complex role of GRAS transcription factors in the arbuscular mycorrhiza symbiosis
Source: Sci Rep. 2019 Mar 4;9:3360. doi: 10.1038/s41598-019-40214-4 (PMC6399340; doi:10.1038/s41598-019-40214-4)
Supplement: Supplementary file 1 — Supplentary Information [file 41598_2019_40214_MOESM1_ESM.pdf]

Insights into the complex role of GRAS transcription factors in the arbuscular mycorrhiza symbiosis

Hartmann, Rico M.[1]; Schaepe, Sieke[1]; Nübel, Daniel[1]; Petersen, Arne C.[1]; Bertolini, Martina[1,2]; Vasilev, Jana[1]; Küster, Helge[1,#]; Hohnjec, Natalija[1,#]

[1] Unit IV-Plant Genomics, Institute of Plant Genetics, Leibniz Universität Hannover, Herrenhäuser Str. 2, D-30419 Hannover, Germany

[2] Department of Food, Environmental and Nutritional Sciences, Università degli Studi di Milano, Via Mangiagalli 25, 20133 Milano, Italy

[#] These senior authors contributed equally to the article

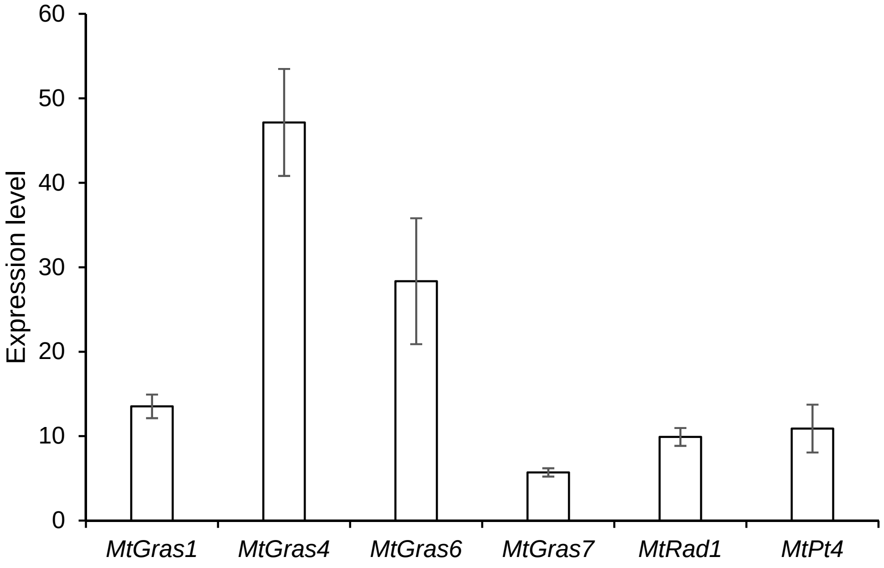

Figure S1: Expression level of selected *MtGras* genes, *MtRad1*, and *MtPt4* in non-mycorrhizal roots under phosphate limitation.

Expression levels base on *Medicago* GeneChip hybridizations reported by Hogekamp *et al.* (2011) and are accessible via the Medicago Gene Expression Atlas (https://mtgea.noble.org/v3/) (Benedito *et al*., 2008). Robust Multiarray Average (RMA) normalized expression levels of three biological replicates derived from plants grown under phosphate limiting conditions (20 µM P) were averaged. n=3, the standard deviation is indicated.

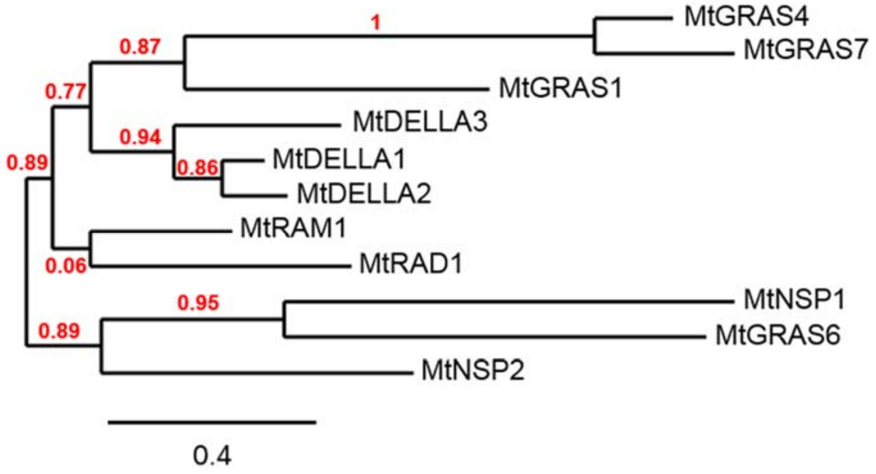

Figure S2: Phylogenetic analysis of selected GRAS TF sequences.
Analyses were performed on the phylogeny.fr platform (Dereeper *et al*., 2008 and 2010). Sequences were aligned using MUSCLE (v3.8.31) (Edgar, 2004), gaps were removed by Gblocks (v0.91b) (Castresana, 2000), and trees were constructed using the maximum likelihood method in PhyML (v3.1/3.0 aLRT) (Guindon *et al*., 2010). Bootstrap values are indicated. Except of the MtGras proteins listed in table 1, the GRAS TF sequences included MtRAM1 (Park *et al*., 2015), MtRAD1 (Rey *et al*., 2017), MtNSP1 (Delaux *et al*., 2013), MtNSP2 (Maillet *et al*., 2011), and the three MtDELLA TFs MtDELLA1, MtDELLA2, and MtDELLA3 (Floss *et al*., 2013).

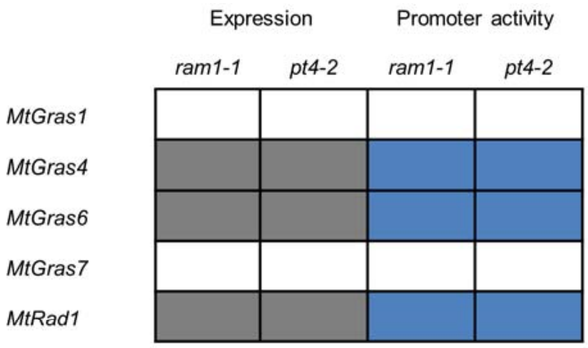

Figure S3: Summarized data on gene expression and promoter activities of GRAS TF genes in *M. truncatula ram1-1* and *pt4-2* mutants.
Significantly reduced (white) or unaffected (grey) transcript levels after *GiTefa*-normalization (Figures 2 and 4) as well as absent (white) or still detectable (blue) promoter activities (Figures 3 and 5) in *ram1-1* and *pt4-2* roots displaying a premature arbuscule degeneration phenotype.

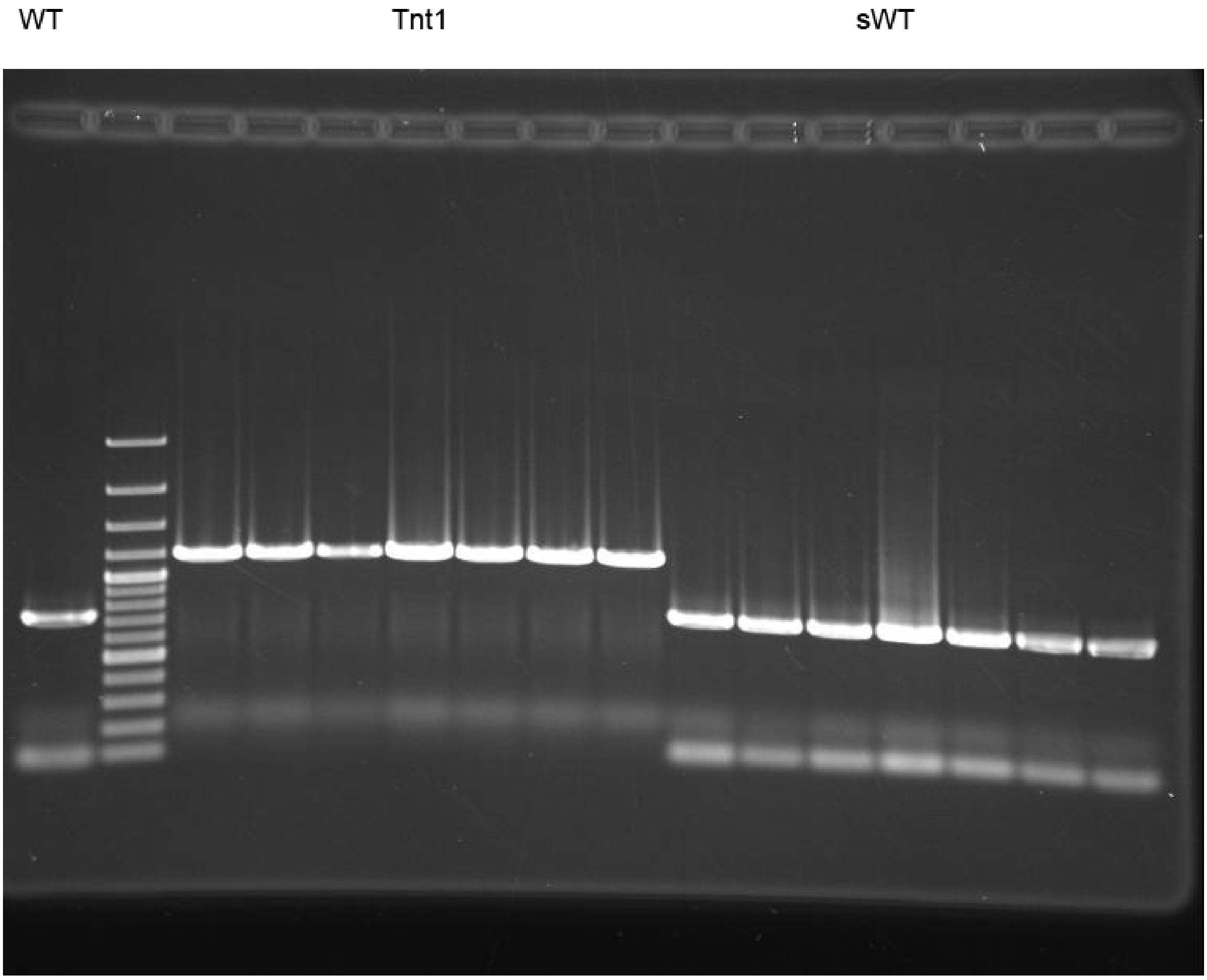

Figure S4: Original, uncropped image of the gel shown in Figure 6B.

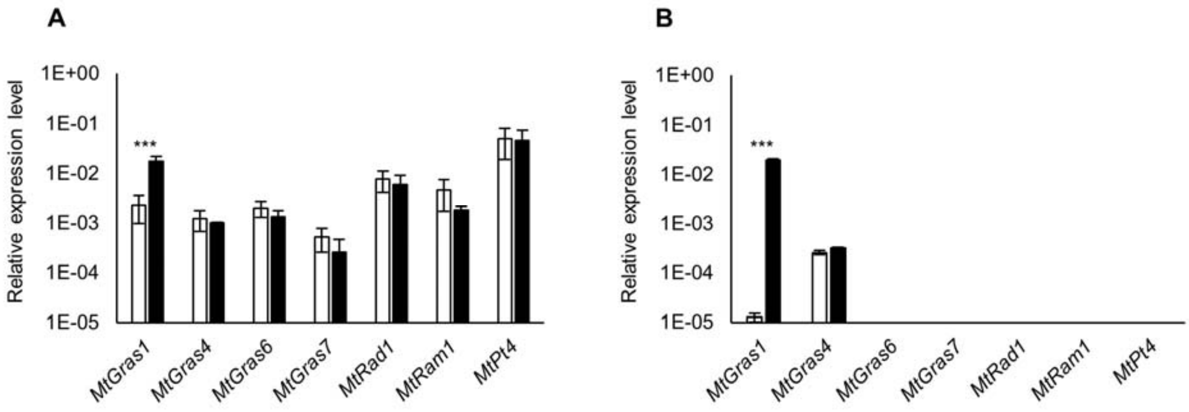

Figure S5: Relative expression of *MtGras1* and selected AM marker genes.
A Mycorrhizal (35 dpi) pPt4:*MtGras1* (black) vs. pPt4:*gusA*int (white) roots. **B** Uninoculated pUbi3:*MtGras1* (black) vs. pUbi3:*gusA*int (white) roots. Transcript amounts are shown relative to *MtTefα*. Roots were harvested at 35 days post inoculation with *R. irregularis*. n=4 biological replicates, error bars represent standard deviations.

Supplemental Table S1
Nomenclature of AM-related genes encoding GRAS TFs.

Gene	Genome ID	MtGEA ID	GRAS subgroup	Alternative IDs
MtGras1	Medtr3g022830	Mtr.7264.1.S1_at	GRASM1*/SCL3**	TF80***/MtGRAS24**
MtGras4	Medtr7g109580	Mtr.1484.1.S1_at	GRASM6*/MIG1****	MtGRAS59**
MtGras6	Medtr2g089100	Mtr.47463.1.S1_at	SHR**/****	MtGRAS12**
MtGras7	Medtr1g086970	Mtr.24642.1.S1_at	GRASM6*/MIG1/SCR**	MtGRAS4**
MtRad1	Medtr4g104020	Mtr.36004.1.S1_at	GRASM5*/Os4****/DELLA**	MtSymSCl3/MtGRAS43**
MtRam1	Medtr7g027190	Not represented	GRASM5*/DELLA**	MtGRAS54**

Identifiers (IDs) in the *Medicago truncatula* genome (Tang *et al*., 2014), the Medicago Gene Expression Atlas (MtGEA; Benedito *et al*., 2008), as well as subgroups and alternative identifiers from the literature are listed. *, Song *et al*. (2010); **, Zhang *et al*. (2017); ***, Park *et al*. (2015); ****, Heck *et al*. (2016).

Supplemental Table S4
Expression of AM marker genes in mycorrhizal *M. truncatula* A17 RNAi:*MtGras1* or *ram1-1* roots in comparison to control roots.

Transcript Cluster ID	Mt gene name	Mt genome 4.0 ID	Annotation	Wild type log2 intensity	ram1-1 log2 intensity	Fold change (wild type vs. ram1-1)	p-value (wild type vs. ram1-1)	gusAint control log2 intensity	RNAi:MtGras1 log2 intensity	Fold Change (gusAint control vs. RNAi:MtGras1)	p-value (gusAint control vs. RNAi:MtGras1)
TC0100003992.mtr.1	MtPt4	Medtr1g028600.1	Phosphate transporter	12,1	2,6	688,8	0,000	12,3	11,4	1,9	0,108
TC0400004806.mtr.1	MtGlp1	Medtr4g052770.1	Germin-like protein	10,9	2,7	288,6	0,000	11,7	10,5	2,3	0,062
TC0700001334.mtr.1	MtMyb1	Medtr7g068600.1	Myb transcription factor	10,2	2,9	163,4	0,000	8,0	8,4	-1,3	0,818
TC0500000972.mtr.1	MtLec5	Medtr5g031030.1	Lectin	9,0	2,5	91,7	0,000	9,9	7,2	6,7	0,030
TC0100001222.mtr.1	MtRam2	Medtr1g040500.1	Glycerol-3-phosphate acyltransferase	10,3	4,0	78,6	0,000	10,3	9,0	2,4	0,099
TC0800000282.mtr.1	MtDef2	Medtr8g012835.1	Defensin	8,4	2,3	65,9	0,000	8,7	7,3	2,7	0,348
TC0800004220.mtr.1	MtTi1	Medtr8g059790.1	Kunitz type trypsin inhibitor	8,9	2,9	64,1	0,000	8,4	8,1	1,3	0,363
TC0500005070.mtr.1	MtGst1	Medtr5g076900.1	Glutathione S-transferase	9,5	3,5	62,1	0,000	9,9	8,6	2,5	0,096
TC0800000278.mtr.1	MtDef1	Medtr8g012805.1	Defensin	8,8	2,9	59,3	0,000	8,8	7,0	3,3	0,240
TC0800002929.mtr.1	MtHa1	Medtr8g006790.1	Plasma membrane H+ATPase	9,6	4,5	34,9	0,000	10,1	8,6	2,9	0,038
TC0800002245.mtr.1	MtNip1	Medtr8g087710.1	Major intrinsic protein (MIP) transporter	9,5	4,5	31,5	0,000	8,5	8,3	1,1	0,950
TC0500003253.mtr.1	MtSbtM1	Medtr5g011320.1	Subtilisin-like serine protease	9,2	4,2	30,8	0,000	10,5	9,0	2,8	0,044
TC0500000966.mtr.1	MtStr2	Medtr5g030910.1	ABC transporter	8,9	4,0	29,1	0,000	9,6	7,7	3,7	0,059
TC0700000122.mtr.1	MtErf1	Medtr7g009410.1	ERF transcription factor	7,6	2,7	28,4	0,000	7,7	6,9	1,8	0,109
TC0800001689.mtr.1	MtLec7	Medtr8g068030.1	Lectin	9,7	5,3	20,0	0,000	11,1	9,4	3,2	0,028
TC0500004795.mtr.1	MtPt8	Medtr5g068140.1	Phosphate transporter	6,2	2,2	15,6	0,000	5,0	3,2	3,4	0,028
TC0700005059.mtr.1	MtBcp1	Medtr7g086190.1	Blue copper protein	7,8	4,6	8,9	0,001	9,3	7,7	3,0	0,033
TC0600000634.mtr.1	MtVapyrin	Medtr6g027840.1	Ankyrin repeat protein	9,0	5,9	8,1	0,000	9,4	7,7	3,1	0,080
TC0300005385.mtr.1	MtScp1	Medtr3g079590.1	Serine carboxypeptidase	10,6	7,9	6,8	0,001	11,8	10,3	2,9	0,032
TC0800003834.mtr.1	MtAnn2	Medtr8g038220.2	Annexin	7,6	5,2	5,4	0,000	8,5	7,2	2,5	0,059
TC0800003833.mtr.1	MtAnn1	Medtr8g038210.2	Annexin	6,8	4,7	4,2	0,000	7,6	7,0	1,5	0,203
TC0700002896.mtr.1	MtAmt1	Medtr7g115050.1	Ammonium transporter	4,8	3,0	3,4	0,002	6,4	4,4	3,9	0,022
TC0200005086.mtr.1	MtCbf1	Medtr2g081600.1	Nuclear transcription factor Y	4,6	2,9	3,2	0,001	6,4	4,2	4,6	0,119
TC0200005087.mtr.1	MtCbf2	Medtr2g081630.1	Nuclear transcription factor Y	4,8	3,3	2,8	0,007	6,6	4,2	5,3	0,160
TC0700003611.mtr.1	MtRam1	Medtr7g027190.1	GRAS transcription factor	8,1	2,7	43,2	0,000	8,9	6,9	4,2	0,042
TC0300003722.mtr.1	MtGras1	Medtr3g022830.1	GRAS transcription factor	6,9	2,7	17,4	0,000	6,5	4,7	3,4	0,004
TC0400002717.mtr.1	MtRad1	Medtr4g104020.1	GRAS transcription factor	7,2	4,4	6,7	0,001	7,9	4,9	8,0	0,035
TC0100005775.mtr.1	MtGras7	Medtr1g086970.1	GRAS transcription factor	4,6	2,6	3,9	0,002	4,2	3,3	1,9	0,044
TC0800003959.mtr.1	MtTF124	Medtr8g442410.1	GRAS transcription factor	6,2	5,4	1,8	0,002	7,2	5,4	3,5	0,027
TC0200002452.mtr.1	MtGras6	Medtr2g089100.1	GRAS transcription factor	5,7	4,4	2,6	0,002	6,8	4,7	4,3	0,041
TC0700005897.mtr.1	MtGras4	Medtr7g109580.1	GRAS transcription factor	4,5	3,3	2,3	0,004	4,9	4,9	1,0	0,653

Supplemental Table S5
Primers for cloning reporter gene, RNAi, overexpression fusions, and genotyping a *Tnt1* line.

Reporter gene fusions

pMtGras1_for	AAAGCATGCAAGCGATGAAAGGGTGTTGAA
pMtGras1_rev	AAACCCGGGGGTGATTTATCTTGAAGAATT
pMtGras4_for	AAAGAATTCCTTGAACCATTATTGGATTA
pMtGras4_rev	AAAAAGCTTATGACGGATTACAAGAGAAA
pMtGras6_for	AAAGCATGCCATTGTACGTAGAGTTCTGA
pMtGras6_rev	AAAAAGCTTGGTTGAAGGAGATTGGAAAA
pMtGras7_XhoI_for	AAACTCGAGCGAATATGCAAAATTGATCC
pMtGras7_HindIII_rev	AAAAGCTTGTTGTTATTGCAAAGAAACTG
pMtRad1_for	AAAGAATTCTCATGAAATTGTTATACAAA
pMtRad1_rev	AAAAAGCTTTGACATATTAATTTCTCAATT

MtGras1 overexpression

MtGras1oex_for	AAAACCCGGGGATGGATTCAGGTTCGCCATAT
MtGras1oex_rev	AAAACCCGGGTCACCTAAATTTCCAGGCCGA

MtGras1 RNA interference

RNAi_Gras1_for	GGGGACAAGTTTGTACAAAAAAGCAGGCTGCTGAGGGAATTGAGATATG
RNAi_Gras1_rev	GGGGACCACTTTGTACAAGAAAGCTGGGTATCAATAATATGCACCACCT

Tnt1 genotyping

NF4813_16_for	AAGTGGGGTCCAGTGCATAG
NF4813_16_rev	TACATTGGAGTCCCCTTCCA
Tnt1-F	GCATTCAAACTAGAAGACAGTGCTACC

Supplemental Table S6
Primers used in real-time RT-PCR experiments.

Primer	NCBI accession	Sequence
MtGras1_for	XM_003598826	TTTAGGCCCTTACAAGATTAC
MtGras1_rev		CTTCACTGAAATAAGTGACCA
MtGras1_oex_for	XM_003598826	TGGATGTAATTTAACGGAGAG
MtGras1_oex_rev		CTTCACTGAAATAAGTGACCA
MtGras4_for	XM_003625927	AGGGTTAAGAAATATTGTTGC
MtGras4_rev		TAAACACATGCCTCACTAAAC
MtGras4_Tnt1_3'_for	XM_003625927	ATAGAATGGTGGAAACTAGGT
MtGras4_Tnt1_3'_rev		CCAAGCTGAGATTGAATACAT
MtGras6_for	XM_003597004	AAGCTTTGGTTATAAATTGCC
MtGras6_rev		GTCCTATGTCAGATTCAAACT
MtGras7_for	XM_003591347	AAACCATAATTCACCTTTGTT
MtGras7_rev		CAGATAATATTGCTTCCATCA
MtRad1_for	XM_003608835	GTGAGGTAGATGAAAATCCAT
MtRad1_rev		GGATAGTTACGGTTGATCTTT
MtMyb1_for	XM_01359369	TACTGCCAAATTTCTGTTCTA
MtMyb1_rev		GGATTGTGTTTTAAAGGATTC
MtPt4_for	AY116211	TCGCGCGCCATGTTTGTTGT
MtPt4_rev		GCGAAGAAGAATGTTAGCCC
GiTefα_for	XM_003588773	ATCCCAAATTTGTTAAGTCTG
GiTefα_rev		ACAGCAACAGTCTGTCTCATA
Giα-Tub_for	GW088233	TGTCCAACCGGTTTTAAAGT
Giα-Tub_rev		AAAGCACGTTTGGCGTACAT
MtRam1_for	XM_003622047	CATTACTACTCCGCAATTTTC
MtRam1_rev		CAACAAACAACCTTTATCCTC

References only mentioned in the Supplementary Information

Dereeper, A. *et al.* Phylogeny.fr: robust phylogenetic analysis for the non-specialist. *Nucleic Acids Research* **36,** W465 (2008).

Dereeper, A. *et al.* SNiPlay: a web-based tool for detection, management and analysis of SNPs. Application to grapevine diversity projects. *BMC Bioinformatics* **12,** 134 (2011).

Edgar, R. C. MUSCLE: multiple sequence alignment with high accuracy and high throughput. *Nucleic Acids Research* **32,** 1792-1797 (2004).

Castresana, J. Selection of Conserved Blocks from Multiple Alignments for Their Use in Phylogenetic Analysis. *Molecular Biology and Evolution* **17,** 540-552 (2000).

Guindon, S. *et al.* New algorithms and methods to estimate maximum-likelihood phylogenies: assessing the performance of PhyML 3.0. *Systematic Biology* **59,** 307-321 (2010).

Delaux, P. M., Bécard, G. & Combier, J. P. NSP1 is a component of the Myc signaling pathway. *New Phytologist* **199,** 59-65 (2013).

Maillet, F. *et al.* Fungal lipochitooligosaccharide symbiotic signals in arbuscular mycorrhiza. *Nature* **469,** 58-63 (2011).
